# Interfacial Electric Fields in Water Nanodroplets Are Weakly Dependent on Curvature and pH

**DOI:** 10.1021/jacs.6c09391

**Published:** 2026-10-08

**Authors:** Gabriele Amante, Fortunata Panzera, Pengchao Zhang, Gabriele Centi, Jing Xie, Ali Hassanali, A. Marco Saitta, Giuseppe Cassone

**Affiliations:** † Department of Chemical, Biological, Pharmaceutical, and Environmental Sciences, 18980 University of Messina, Viale F. Stagno d’Alcontres 31, Messina 98166, Italy; ‡ Institute for Chemical-Physical Processes, 9327 National Research Council (CNR-IPCF), V.le F. Stagno d’Alcontres 37, Messina 98158, Italy; § Laboratoire de Physique de L’école normale supérieure de Paris, CNRS, 27063 École Normale Supérieure Université Paris Sciences et Lettres, Sorbonne Université, Université de Paris, Paris 75005, France; ∥ Center for Combustion Energy, Department of Energy and Power Engineering, and Key Laboratory for Thermal Science and Power Engineering of Ministry of Education, 12442 Tsinghua University, Beijing 100084, China; ⊥ Ministry of Education Key Laboratory of Cluster Science, Beijing Key Laboratory of Photoelectronic/Electrophotonic Conversion Materials, School of Chemistry and Chemical Engineering, 47833 Beijing Institute of Technology, Beijing 100081 P. R. China; # 18473 International Centre for Theoretical Physics (ICTP), Strada Costiera 11, Trieste 34151, Italy; ■ Institut Universitaire de France (IUF), https://www.iufrance.fr/

## Abstract

The origin of enhanced reactivity in aqueous microdroplets remains unresolved, with interfacial electric fields (IEFs) frequently proposed as catalytic drivers. Here, we present a quantum-mechanical, spatially resolved characterization of the electric field at air-water interfaces by combining deep learning molecular dynamics with *ab initio* re-sampling. Across planar interfaces and nanodroplets spanning a range of curvatures and charge states, we consistently identify an outward-directed field of ∼1.0–1.2 V/Å along the intrinsic surface normal. Notably, the field magnitude scales linearly with the average number of hydrogen bonds per interfacial molecule, directly linking the IEF to the local hydrogen-bond network. Strikingly, curvature and pH exert only a moderate influence on the IEF, with differences becoming negligible at experimentally relevant droplet sizes and pH regimes. Accordingly, the reactivity enhancements observed in *μ*m-sized droplets cannot be attributed to variations in the IEF, which changes by only ∼10^–5^ between 3 and 40 μm-sized droplets. Furthermore, the IEF is highly localized within the interfacial region, decaying over just a few Å. This pronounced spatial confinement ties the field to the local electronic structure, establishing the IEF as an intrinsic interfacial property rather than an independent mechanistic driver of “on-water” catalysis.

## Graphical abstract

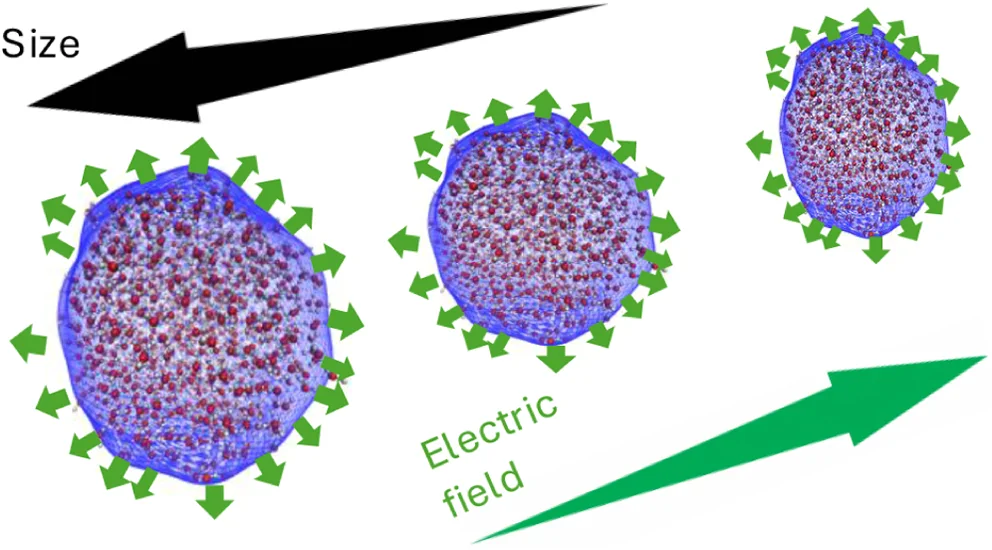

## Introduction

Over the past decade, the chemistry of water microdroplets has attracted significant attention, as reactions within them often proceed along pathways and rates markedly different from bulk aqueous solutions.^1–4^ Experiments have shown that processes such as urea synthesis from CO_2_ and NH_3_,^5^ sulfate oxidation,^6^ and ammonia formation from N_2_ ^7^ can occur under ambient conditions with unexpectedly high efficiencies. These findings underpin the concept of “on-water” catalysis, whereby the air-water interface provides a chemically distinct environment that promotes chemical reactions unfavorable in the bulk.^8^

Understanding the molecular origins of such behavior remains an open challenge.^9^ The droplet interface combines several features absent in homogeneous aqueous systems, including partial solvation, strong density gradients, and enhanced molecular anisotropy. Among the mechanisms proposed to rationalize microdroplet reactivity, particular attention has been devoted to the role of electric fields at aqueous interfaces,^10^ these latter originating from the termination of condensed matter at the vacuum and the resulting electronic charge distribution spilling over into that vacuum.^11,12^ Several studies have suggested that the air-water boundary may sustain electric fields on the order of ∼0.2 V/Å,^6,9,13–15^ a magnitude that could potentially influence chemical reactions by stabilizing charge-separated states or lowering activation barriers. This hypothesis conceptually draws support from numerous computational^16–24^ and experimental^25–27^ studies demonstrating that externally applied and *adequately oriented static* electric fields can profoundly modify chemical reactivity in both homogeneous^18,28^ and heterogeneous^29^ aqueous environments. Although the *conditio sine qua non* for catalysis is that the field must be aligned with the reaction axis, spontaneous electrostatic fields generated at the droplet interface have been proposed to act as catalytic driving forces for microdroplet chemistry.^6,7,9,14,30–32^

Droplet curvature has also very recently been invoked as a potential contributor to interfacial electrostatic interactions. Because decreasing droplet size modifies the properties of surface water molecules, several works have proposed that smaller droplets may amplify interfacial electric fields (IEFs) and alter the local electrostatic environment at the surface, thereby increasing the catalytic properties of air-water interfaces.^33,34^ In addition, interfacial water exhibits complex acid-base and charge-transfer properties that can lead to non-trivial pH and ion distributions across the air-water boundary.^35–38^ The behavior of ions at aqueous interfaces is itself subtle: specific-ion effects arising from differences in hydration, size, and polarizability can significantly perturb interfacial structure and electrostatics.^39–41^ At the same time, the possible involvement of reactive oxygen species, ozone contamination, or droplet charging processes complicate the interpretation of microdroplet experiments.^8,9,35^

Consistently accurate quantum-chemical calculations indicate that electric fields of ∼0.2 V/Å only modestly perturb charge transfer in hydrogen bonds^42^ and redox energetics.^43^ Earlier classical electrostatic simulations also concluded that enhanced interfacial reactivity cannot be explained solely by electric field magnitude.^15^ Recent vibrational sum-frequency generation (SFG) measurements by Bonn and coworkers at the planar air-water interface revealed no spectroscopic signatures consistent with exceptionally strong electric fields.^44^ Besides, classical molecular dynamics simulations by Hassanali and coworkers demonstrated that the electric field experienced by the phenol O–H group at the interface is essentially governed by its immediate hydration environment.^45^ Bonn and Hassanali show that the IEF displays no unique features compared to that in the bulk.

Despite these important advances, obtaining reliable estimates of the IEFs remains challenging. Many theoretical approaches rely on classical electrostatic models with fixed charges, thereby neglecting polarization effects, while spectroscopic studies often infer field strengths only indirectly via Stark frequency-field correlations.^46^ Here, we address these limitations by combining neural-network-based deep-learning molecular dynamics with *ab initio* resampling, enabling an accurate and spatially resolved quantum-mechanical characterization of the electric field at both flat and curved air-water interfaces in nanodroplets of varying size under neutral and charged conditions. Contrary to common expectations, we find that curvature and pH exert only a minor influence on the IEFs, indicating that the enhanced reactivity of these environments must originate from alternative molecular mechanisms.

## Methods

Deep Potential Molecular Dynamics (DPMD) simulations of air-water systems were carried out with the LAMMPS software (v. 29Aug2024).^47^ We employed the previously published short-range reactive DP model of Zhang and Xu,^48^ which was trained to reproduce DeePKS-generated reference data with approximate M06-2X^49^ accuracy. For more details, the interested reader can refer to the dedicated repository for information on the training dataset and relevant validation.^50^ As detailed in the Supporting Information (SI), a new committee model analysis was performed to validate the DP model against isolated nanodroplets, showing similarly small deviations of the planar air-water interfaces already validated in Ref 48. Furthermore, as reported in Figure S3 and Table S1 of the Supporting Information, a validation analysis on small systems against M06-2X reference data is indicative of the quality of the adopted DP model. This DP model is short-ranged, potentially leading to artifacts.^51^ However, tests performed on the behavior of the water dipoles in the bulk interiors of all nanodroplets—including those with excess ions—as well as benchmarks against MB-pol(2023)^52^ do not evidence any long-distance, bulk-spanning dipolar alignment, identified as a characteristic artifact of short-range potentials, as discussed in the SI. Besides, as reported in Figure S19 of the Supporting Information, a direct validation of the DP model against a long-range DPLR model^53^ for the main systems simulated here reveals very small associated errors, highlighting once again the reliability of the employed model.

Periodic simulations were carried out to mimic the behavior of the flat air-water interface, whereas non-periodic simulations of isolated nanodroplets were performed to probe the impact of interfacial curvature on the surface electric field. As for the flat interface, samples composed of 256 and 512 H_2_O molecules were considered, with box edges of *a* = *b* = 19.641 Å, *c* = 45.000 Å and *a* = *b* = 24.830 Å, *c* = 60.000 Å, respectively. Seven isolated nanodroplets composed of 256, 384, 512, 640, 768, 896, and 1024 H_2_O molecules were simulated in cubic boxes with edges of 44, 48, 60, 63, 65, 67, and 68 Å, respectively. The respective radii *R* and the curvature *κ* of each droplet are listed in Table S2 of the Supporting Information. The charged nanodroplets composed of 507 H_2_O and 5 OH^–^ (*q* = −5*e* ^–^), 511 H_2_O and 1 OH^–^ (*q* = -*e* ^–^), 511 H_2_O and 1 H_3_O^+^ (*q* = +*e* ^–^), and 507 H_2_O and 5 H_3_O^+^ (*q* = +5*e* ^–^) were simulated in the absence of any compensating uniform background charge, in addition to a globally neutral nanodroplet composed of 502 H_2_O, 5 OH^–^, and 5 H_3_O^+^ which, however, underwent charge recombination. Inclusion of the ions has been performed randomly within the surface region. Each DPMD simulation was carried out in the NVT canonical ensemble for at least 1 ns. The temperature was kept fixed at 300 K via the canonical-sampling-velocity-rescaling (CSVR) thermostat^54^ with a damping factor of 0.04 ps.

500 randomly chosen configurations were extracted from all DPMD trajectories and resampled via *ab initio* calculations at the PBE+D3 ^55–57^ Density Functional Theory (DFT) level to obtain the electric field **E**(**r**) directly from the electron density *ρ*_*e*_(**r**), within a pseudopotential description of the nuclei, via Poisson’s equation:

$$\nabla^{2}\phi \left(r\right)=-4\pi \rho_{e}\left(r\right),,E\left(r\right)=-\nabla \phi \left(r\right)$$

where *ϕ*(**r**) is the electrostatic potential. Although GGA exchange-correlation functionals are affected by the self-interaction error, which tends to overdelocalize electronic charge, especially along hydrogen bonds,^58^ its impact on the present analysis is quantitatively negligible in the regime of interest. In particular, the interfacial electric fields (IEFs) reported here are intrinsically large (on the order of ∼1 V/Å), such that any residual error associated with density delocalization is substantially smaller than the variations we discuss. This assessment is directly supported by benchmark calculations performed at the SCAN functional^59^ and r2SCAN functional^60^ levels on air-water nanodroplets, which yield essentially indistinguishable IEF distributions (see Figure S15 of the Supporting Information). Taken together, these results demonstrate that the magnitude and trends of the interfacial fields are robust with respect to the underlying density functional approximation, and that delocalization errors do not affect the physical conclusions drawn in this work. Due to their non-periodic nature and the large size of the vacuum regions, SCAN and r2SCAN calculations of nanodroplets containing more than 128 H_2_O species were not computationally feasible on computing nodes with 384 GB RAM. Electronic structure calculations were carried out by employing the CP2K software (v. 2024.3).^61^ Wavefunctions of the atomic species have been expanded in TZVP basis sets with Goedecker-Teter-Hutter (GTH) pseudopotentials using the GPW method.^62^ For the PBE+D3 calculations, a planewave cutoff of 400 Ry was used, while for the SCAN and r2SCAN a cutoff of 1200 Ry was adopted.

After computing the electric field vector from the total electrostatic potential (electrons+nuclei) via Poisson’s equation on dense spatial grids of meshes in the range ∼0.05–0.15 Å (see Table S4 of the Supporting Information for tests), subsequent analyses were performed using in-house C++ codes. For each configuration extracted from the DPMD simulations, the instantaneous Willard-Chandler (WC) interface was determined from the coarse-grained molecular density following the procedure introduced in Ref 63. The intrinsic interface was then identified as the isodensity surface corresponding to half of the bulk density. Using this surface, the simulation space was partitioned into distinct spatial regions according to the distance from the WC interface. As usual, the depth thresholds for discriminating between the bulk and the air-water interfacial regions were identified via the location of the first pronounced minimum of the density profiles reported in the SI (Figure S4). We did not further divide the interfacial region into a sequence of second, third, and subsequent molecular layers, following our philosophy to minimally constrain the space partitioning and hence the emerging results. In fact, any attempt of localizing a quantity that is by definition non-local—such as the continuous electron density and the corresponding electric field —introduces artifacts. For the same reason, we did not exclude from the field evaluation spatial regions around the nuclei, a procedure often undertaken when employing classical point charges, leading to local field divergences. For each grid point of the electric field, the corresponding normal vector to the intrinsic surface, ***n***_int_, was computed. The electric field vector ***E*** = (*E*_*x*_,*E*_*y*_,*E*_*z*_) was then projected onto the local WC reference frame, and the normal component of the electric field was obtained as *E*_*n*_ = ***E*** · ***n***_int_. Finally, the projected field components were accumulated over all grid points belonging to the same spatial region, allowing us to compute the average electric field along the interface normal in the bulk, interfacial, and vapor regions. A field so defined is not a molecule-resolved field, a field evaluated at a particular reactive site, or the projection of the field along a chemical reaction coordinate, but explicitly takes into account its intrinsic non-local nature. For the sake of clarity, the reported electric field strengths refer exclusively to the projection along the WC surface normal and therefore cannot be directly compared with other definitions of the electric field. Nevertheless, their magnitude (∼1 V/Å) is fully consistent with independent estimates obtained from the electrostatic potential and the corresponding experimentally accessible mean inner potential, as shown in the SI (Figure S14) and reported in previous studies.^11,12^

## Results and Discussion

### Structural and Electronic Properties of Air-Water Interfaces

The microscopic behavior of H_2_O molecules at the air-water boundary differs markedly from that in the bulk. While molecular orientations are isotropic in the bulk, pronounced anisotropies arise at the liquid-vapor interface.^64^ Additional changes may occur with increasing curvature *κ* = R^–1^—from planar slabs to nanodroplets of decreasing size. Figure 1a reports the orientational distributions P­(cos*α*) of water dipole moments relative to the instantaneous Willard-Chandler (WC) surface normal, for both interfacial and bulk molecules. Whereas bulk regions exhibit nearly uniform distributions, as expected, distinct orientational populations emerge at the interface.

**Figure 1:**
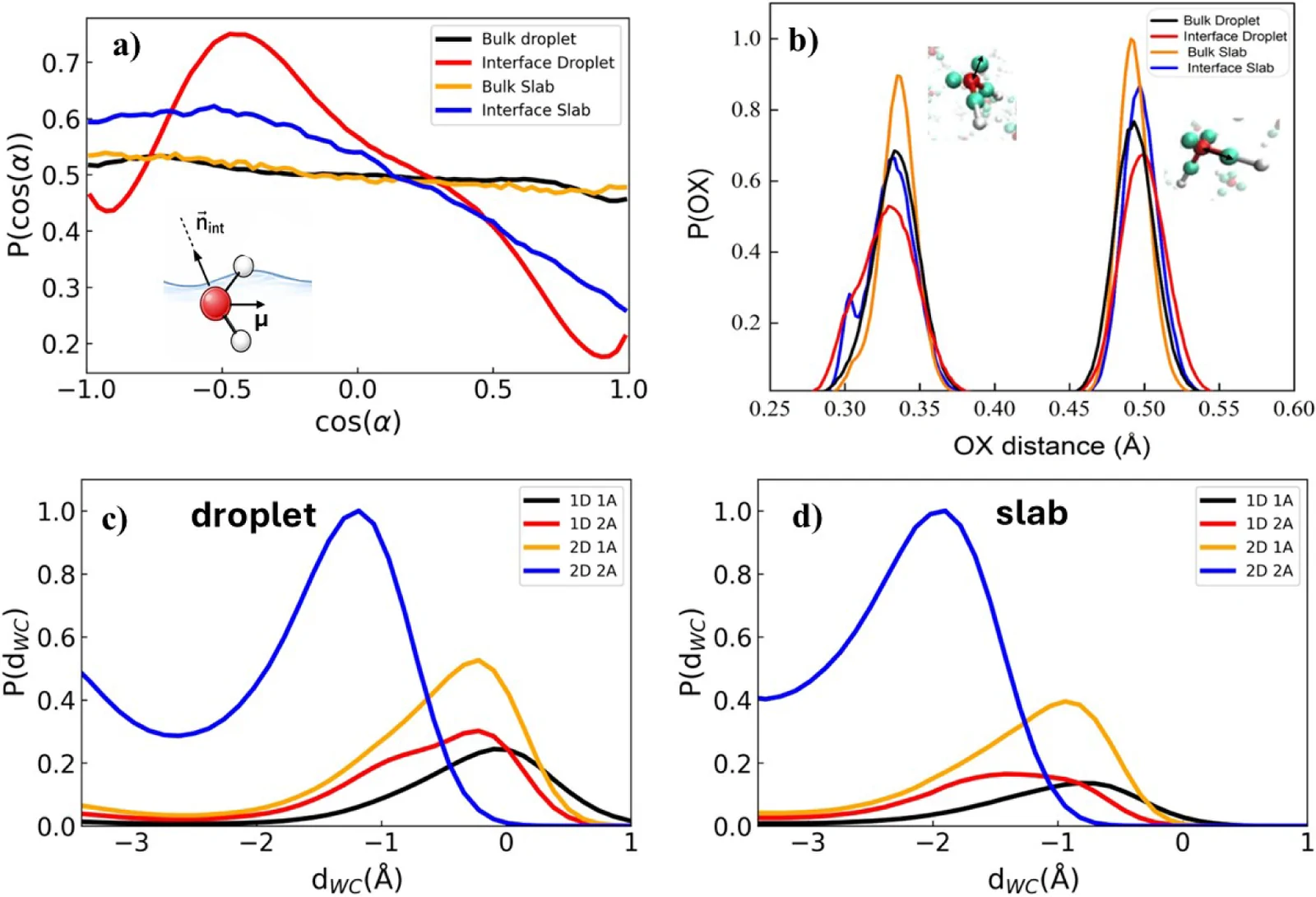
Orientation distributions of molecular dipole moments P­(cos*α*) (a) with respect to the Willard-Chandler surface normal, determined in the bulk and at the interfaces of a nanodroplet and a flat slab system composed of 512 H_2_O molecules (see legend). In the respective inset, involved vectors are illustrated. Panel (b) reports the distribution of the O-X distance, where X denotes the position of the Maximally Localized Wannier Functions centers (visualized in cyan in the insets), for bulk and interfacial molecules in the droplet and slab geometries (see legends). Panels (c) and (d) show the spatial distribution of selected hydrogen-bond donor-acceptor (D-A) families as a function of the distance from the instantaneous Willard-Chandler interface *d*_WC_. Curves correspond to different hydrogen-bond topologies: 1D-1A (black), 1D-2A (red), 2D-1A (orange), and 2D-2A (blue). See Figure S9 of the Supporting Information for the wider spectrum of hydrogen-bonded molecular families.

In the negative cos*α* region, interfacial dipoles preferentially point toward the liquid phase, reflecting asymmetric cohesive interactions. The distribution maximum displayed in Figure 1a around cos*α* ∼ −0.45 indicates configurations that tend to maximize hydrogen bonding. In the positive cos*α* region, corresponding to dipoles pointing toward the vapor phase, the slab distribution decays monotonically, whereas the droplet shows a shoulder around cos*α* ∼ 0.25. This feature arises from configurations in which one OH bond remains oriented toward the liquid, stabilizing partially outward-pointing dipoles. In fact, by inspecting the orientations of the OH covalent bonds identified by the angle *β* formed with the WC surface normal, as reported in Figure S6b of the Supporting Information, a similar shoulder appears at cos*β* ∼ −0.25. Thus, a partial orientation of the dipole moment toward the vapor phase (*α* ∼ 75^◦^) is stabilized by one of the OH bonds of the same molecule pointing toward the interior of the liquid phase (*β* ∼ 105^◦^).

Overall, the air-water interface of curved nanodroplets exhibits a more heterogeneous population of molecular orientations than flat interfaces, reflecting the larger number of H-bonds formed by water molecules in the planar slab, an aspect that will be discussed later. In the same spirit, de la Puente and Laage^64^ have recently reported a larger number of free OH groups in a small nanodroplet of 128 H_2_O with respect to a flat slab. Nevertheless, the fractions of outward- and inward-oriented molecules remain nearly identical in the droplet and flat slab (Figure S6c,d of the Supporting Information). The excess of dipoles pointing toward the bulk, leading to an average *α* ∼96^◦^ in the droplet (*α* ∼ 94^◦^ in the slab), suggests the presence of a net electric field directed outward from the liquid toward the vapor. As discussed in several important works,^11,12^ the natural termination of the liquid phase and the associated spill over of the electron density into the vacuum create an interfacial field, as also shown in Figure S14 of the Supporting Information.

The liquid-vapor interface is a strongly anisotropic environment in which the hydrogen-bond network is partially disrupted, progressively destabilizing tetrahedral coordination and leading to polarization states distinct from those in the bulk. One way to quantify the resulting impact on the underlying electronic structure is through the analysis of Wannier functions. Specifically, we computed the Maximally Localized Wannier Function (MLWF)^65^ centers for all water molecules. Figure 1b illustrates the O-X distributions, where X denotes the positions of the MLWF centers and O is the oxygen atom of a given H_2_O. Each water molecule exhibits four MLWF centers: two associated with OH covalent bonds [*d*(*OX*) ∼ 0.5 Å] and two corresponding to lone pairs [*d*(*OX*) ∼ 0.35 Å]. Interfacial molecules display broader and slightly shifted distributions relative to the bulk, indicating increased electronic asymmetry. A distinct population at short O-X distances (∼0.30 Å)—visible in the curved nanodroplet interface (red), but especially evident in the flat slab surface (blue)—signals molecules with reduced hydrogen-bond acceptor capability.

This structural heterogeneity is further elucidated in Figure 1c,d, which report the distribution of hydrogen-bond donor-acceptor (D-A) families as a function of the distance from the WC interface. While the bulk is overwhelmingly dominated by tetrahedral 2D-2A configurations, the interfacial region exhibits a pronounced enrichment of under-coordinated species such as 2D-1A and 1D-2A, reflecting asymmetric and partially disrupted hydrogen-bond networks.^38,66^ Notably, the curved interface of the droplet displays an even higher population of under-coordinated motifs—including 2D-1A, 1D-2A, and 1D-1A—relative to the planar air-water slab (cf. Figure 1c and d). Despite this difference, the qualitative evolution of hydrogen-bonding populations remains consistent across the two interfaces, indicating a common underlying interfacial de-structuring mechanism.

Asymmetries in the hydrogen-bond network manifest directly through subtle differences in electronic polarization between the hydrogen-bond accepting and donating environments of water molecules.^67,68^ To probe this effect, we determined the net charge on each molecule using a recently developed method that partitions the real-space electron density through a Voronoi analysis.^69^ Importantly, the charge-transfer values extracted here arise from intrinsically quantum-mechanical effects associated with both short- and long-range electronic polarization. Specifically, configurations with excess donor bonds tend to become negatively charged, whereas those with excess acceptor bonds acquire a positive charge. Table 1 quantifies this effect for droplet (left) and slab (right) geometries, while the underlying methodology is discussed in SI. It is worth stressing that a negative (positive) mean charge transfer corresponds to an electron depletion (accumulation); hence, red (blue) entries represent positively (negatively) charged H_2_O molecules. A clear trend emerges: hydrogen-bond donor-rich configurations (e.g., 1D-0A, 2D-0A, and 2D-1A) exhibit electron accumulation (blue entries), while acceptor-rich ones (e.g., 0D-1A, 0D-2A, 1D-2A, and 1D-3A) show electron depletion (red entries). Nearly symmetric configurations such as 2D-2A remain essentially neutral, consistent with previous studies.^38,70,71^ Crucially, the close similarity in the charge-transfer trends between droplet and slab geometries indicates that this relationship is largely independent of curvature.

**Table 1:**
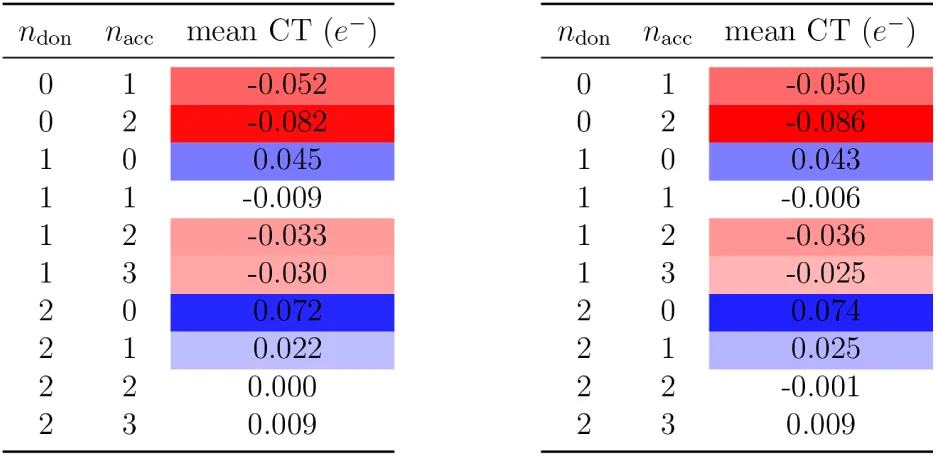
Mean Charge Transfer (CT) Associated with Different Hydrogen-Bond Donor-Acceptor Families for Interfacial Molecules in the Droplet (Left) and Planar Slab (Right) Geometry^a^

a Positive values (blue entries) indicate electron accumulation while negative values (red entries) indicate electron depletion. Negligible CT values are not colored.

An important outcome of the preceding results is that the outermost interfacial layer exhibits a heterogeneous and electronically polarized environment, where coordination defects caused by asymmetries in hydrogen bonding lead to charge transfer. In particular, molecules exposing free OH groups toward the vapor phase tend to carry a positive excess charge, whereas configurations exposing lone pairs toward the vacuum phase correspond to negatively charged H_2_O molecules. The interface thus emerges as a fluctuating ensemble of oppositely charged environments arising from variations in hydrogen-bond topology.

### Curvature Effects on the Interfacial Electric Field

Although the two dominant charged configurations (2D-1A and 1D-2A) populate similar interfacial regions (Figure 1c,d)—thus ensuring overall charge neutrality and precluding any macroscopic capacitive charge separation—the symmetry breaking of the electron density, the preferential orientation of water dipoles toward the liquid phase, combined with locally polarized and charged H_2_O species, generate a finite interfacial electric field (IEF).

Figure 2a reports the average electric field produced by the interfacial region and projected along the local WC surface normal, while the corresponding distributions are displayed in Figure S11a of the Supporting Information. For all systems considered here, the field is positive, indicating an outward orientation from the liquid toward the vapor phase, as also confirmed by the laterally averaged electrostatic potential V­(z) reported in Figure S14 of the Supporting Information. Its magnitude ranges between ∼1.0 and 1.2 V/Å, *i.e.*, about 5–6 times larger than previous classical theoretical estimates.^14^ This difference mainly arises because the electric field, being a vector quantity, depends critically on how it is probed: earlier studies typically projected it along the direction of the OH bond, which is generally less aligned with the local field than the WC surface normal adopted here. Furthermore, we observe, in a dedicated analysis discussed in Figure S13 of the Supporting Information, that dressing water molecules with classical SPC/E charges leads to field estimates along the surface normal that are an order of magnitude lower than those arising from the quantum electron density. Interestingly, the average IEF strength we measure with our method is in line not only with the negative derivative of V­(z) (*i.e.*, E­(z) = −dV­(z)/dz) in the interfacial region displayed in Figure S14b of the Supporting Information, but also with independent estimates from V­(z).^11^ These independently derived results—spanning three field definitions and SPC/E-based re-sampling—show that different constructions yield different field magnitudes but the same outward direction. They also confirm that the main comparative trends are robust to the statistical representation and consistent with previous work. Finally, it is worth remarking that whereas the distributions of the electric field from our methodology are Gaussian-like, those emerging from Ref. 14 are Lorentzian, as also visible from a direct comparison of Figures S11 and S12 of the Supporting Information. Such an intriguing difference will be carefully investigated in future works.

**Figure 2:**
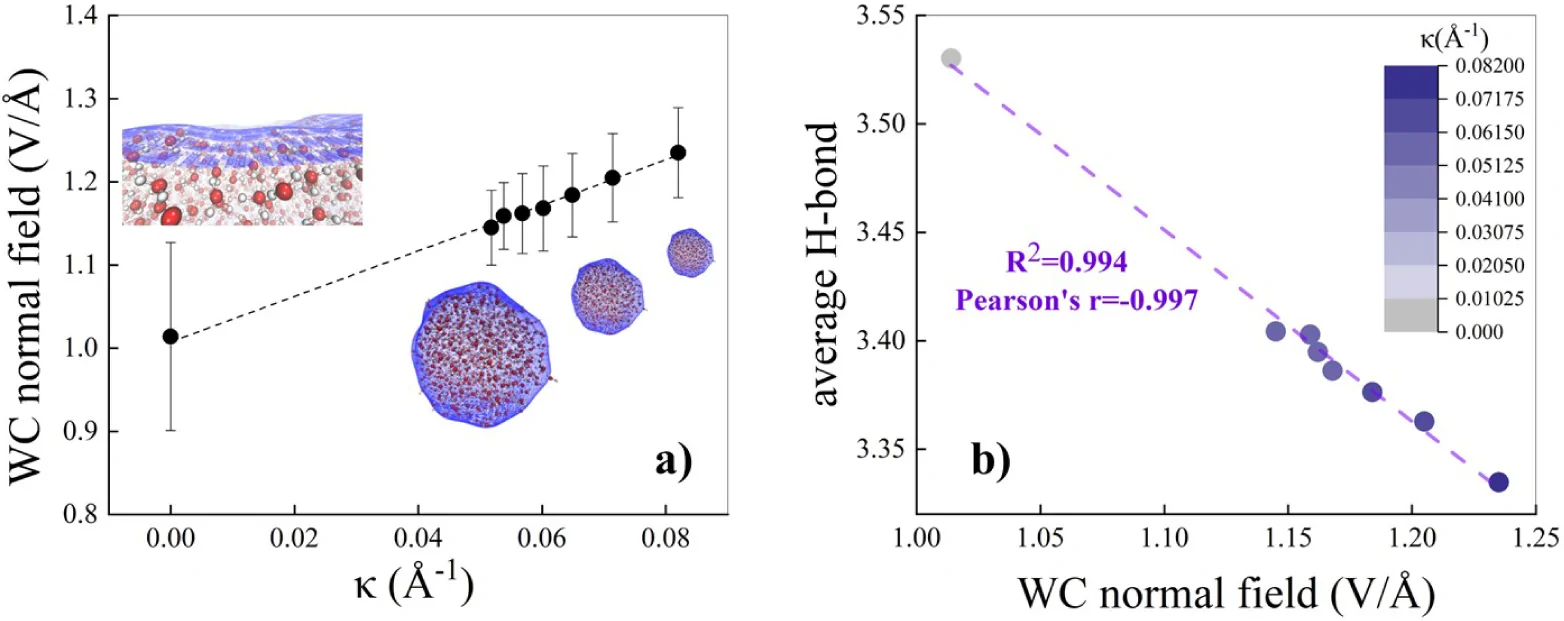
(a) Average interfacial electric field projected along the Willard-Chandler surface normal (with standard deviation used as error bar) as a function of the interface curvature *κ* for droplets of different sizes and for the planar slab limit, whose structures are depicted as insets. (b) Correlation between the interfacial electric field and the average number of hydrogen bonds per interfacial molecule. The color scale indicates the corresponding curvature. Dashed lines are linear fits of the data points.

Figure 2a shows a remarkable linear dependence of the IEF on the curvature of the interface. As the curvature *κ* increases, corresponding to smaller droplets, the magnitude of the field slightly increases. Conversely, as the droplet size increases and the curvature decreases, the field gradually approaches the value observed in the planar slab limit of zero curvature, which represents the infinite-radius case. Although the trend with curvature is systematic, the overall variation of the IEF remains relatively small across the range of droplet sizes considered here. Recent experimental studies have reported significant differences in chemical reactivity in water microdroplets with diameters ranging from approximately 3 to 40 μm and have attributed this effect to variations in the IEF.^33,34^ Our results indicate that, at least from a structural and electrostatic perspective, curvature alone produces only marginal variations in the interfacial field strength in the regime of experimentally probed curvature. Although no computationally feasible approach can simultaneously describe micrometer-sized droplets, non-equilibrium sample evaporation, and charge redistribution processes, extrapolation of the curvature dependence of the IEF to micrometer-sized droplets (3–40 μm) reveals variations on the order of 10^–5^ V/Å, *i.e.*, five orders of magnitude smaller than the field itself. These findings strongly suggest that electric fields cannot be the dominant driving force for the observed enhanced reactivity at microdroplet interfaces.

To gain further insight into the microscopic origin of the IEF, Figure 2b correlates the field intensity with the average number of hydrogen bonds per interfacial molecule. Notice that in our analysis we include all hydrogen bonds and not only those under a specific orientation with respect to the interface. A clear anticorrelation is observed: as the hydrogen-bond network becomes more complete, the magnitude of the electric field decreases. In other words, droplets with lower curvatures up to the slab limit exhibit a more complete hydrogen-bond structure and correspondingly weaker IEFs. This behavior highlights the intimate connection between hydrogen-bond topology and the electrostatic properties of the air-water interface. In highly curved droplets, the hydrogen-bond network is more disrupted, leading to a larger population of under-coordinated molecules, larger polarization and charge transfer phenomena, and hence a stronger net electric field. As the interface becomes progressively flatter, the hydrogen-bond network becomes more structured, and the interfacial field correspondingly weakens. Nevertheless, even in the slab limit, the hydrogen-bond coordination remains lower than in the bulk phase (see, *e.g.*, Table S3 of the Supporting Information), reflecting the intrinsically incomplete hydrogen-bond network of the liquid-vapor interface highlighted in Figure 1.

### pH Effects on the Interfacial Electric Field

Several experimental methodologies exist to produce water microdroplets, some of them giving rise to globally neutral droplets, while others, most notably electrospray ionization (ESI), generate highly charged droplets through the application of strong voltages.^72,73^ Microdroplets can also be produced via gas nebulization,^74^ ultrasonic humidification^75,76^ vapor condensation,^77^ droplet deposition on surfaces,^78^ or levitation techniques.^79^ These methods rely on distinct mechanisms, all involving significant energy input (electric fields, sound, or heat) that perturbs water’s physico-chemical state. ESI can generate droplets with excess H_3_O^+^ or OH^–^ without counterions.^73^ Even without external fields, nebulization and sonication can yield charged droplets via contact electrification or ion partitioning,^9^ thereby shifting the autoprotolysis equilibrium and promoting excess ionic species. Given the catalytic activity of such charged nanodroplets, disentangling the roles of excess charge and IEFs becomes essential.

To quantify the impact of water counterions on the IEF, we simulated several nanodroplets with excess H_3_O^+^ or OH^–^. As detailed in the Methods section, we simulated charge states corresponding to −5*e* ^–^, -*e* ^–^, +*e* ^–^, and +5*e* ^–^, hence probing regimes of 13% and 66% of the Rayleigh limit for Coulomb fission. These charge states approximately correspond to net pH values of 13.74, 13.04, 0.96, and 0.26, respectively. Figure 3a shows that the IEF linearly increases with the total excess charge, suggesting that H_3_O^+^ and OH^–^ ions act effectively as point charges in shaping the interfacial field. Albeit non-neutral systems without counterions cannot be directly linked to macroscopic pH; when viewed on a logarithmic scale one observes some other behavior as a function of the nominal pH, or more generally the excess positive or negative charge (Figure 3b). It turns out that the most significant variations of the electric field occur at extremely acidic or basic pH values. Interestingly, whereas at acidic pH values the IEF increases, in basic conditions it measurably decreases with respect to the neutral case. This observation reconciles with several factors governing the nature of hydronium and hydroxide ions. Firstly, a positively charged ion (H_3_O^+^) localized at the interface becomes an additional field source. On the contrary, an anion (OH^–^) acts as a sink of the electric field lines. Secondly, the different chemical nature of the water counterions leads to a sizably different behavior at the interface. In fact, whereas H_3_O^+^ is a good hydrogen-bond donor (*i.e.*, up to three) but a poor acceptor, the OH^–^ moiety can donate a single weak hydrogen bond but is capable of accepting up to 4 hydrogen bonds, creating over-coordinated solvation environments.^80^ Those features confer different affinities of those ions toward the interface.^39–41^ In particular, hydronium tends to localize in the topmost layer of the droplet, whereas hydroxide exhibits the tendency to reside slightly deeper in layers between the interface and the bulk,^81^ as shown by the distributions of Figure 3c and in the snapshots of Figure 3d,e. In line with other computational evidences stemming from reactive classical force fields,^14^ the emerging picture suggests only modest variation of the IEF achieved at extreme pH values. These findings challenge the idea that the electric field generated by charged droplets might be the source for the observed enhanced catalysis as a function of the applied voltage in, *e.g.*, electrosprayed charged droplets.^73^

**Figure 3:**
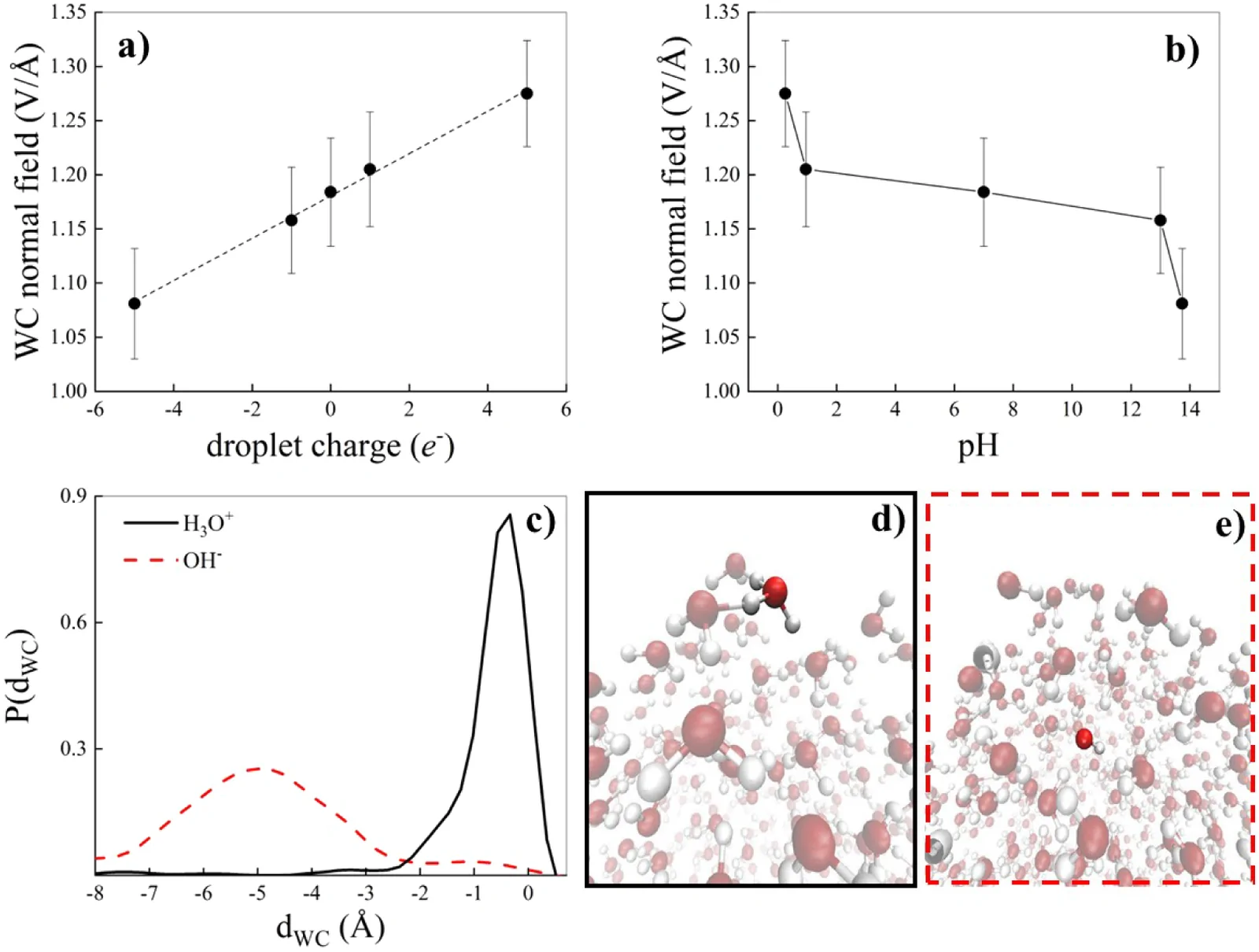
Average interfacial electric field projected along the Willard-Chandler surface normal (with standard deviation used as error bar) as a function of the droplet charge (a) and the resulting pH (b). In panel (a), the dotted line is a linear fit of the data points. In panel (c), the distributions of the distances of an excess hydronium (solid black line) and hydroxide (dashed red line) species with respect to the Willard-Chandler instantaneous surface in nanodroplets containing 511 H_2_O molecules and one excess ion are displayed. Panels (d) and (e) depict typical molecular arrangements of the hydronium and hydroxide water counterions, respectively, extracted from our deep potential molecular dynamics simulations.

### Spatial Extension of the Interfacial Electric Field

Figure 4 reports the distribution of the electric field projected along the WC surface normal across distinct spatial regions relative to the interface, enabling a quantitative assessment of its evolution with distance. Four regions are identified: bulk, interfacial, near-vapor (vapor1), and far-vapor (vapor2). The bulk region extends from the liquid interior up to 2.6 Å and 3.4 Å below the WC instantaneous interface in the droplet and slab systems, respectively. The interfacial region spans from this threshold to 0.8 Å (0.2 Å) above the WC surface in the droplet (slab), thereby including all molecules residing at the interface. This difference arises from the distinct positioning of the WC surface in curved and planar geometries, as also reflected in the density profiles reported in Figure S4 of the Supporting Information. The vapor region is further subdivided into two adjacent layers: vapor1, with a thickness of 2.0 Å (2.6 Å) in the droplet (slab), and vapor2, 1.2 Å thick in both systems, both located above the interface, as illustrated in the insets of Figure 4.

**Figure 4:**
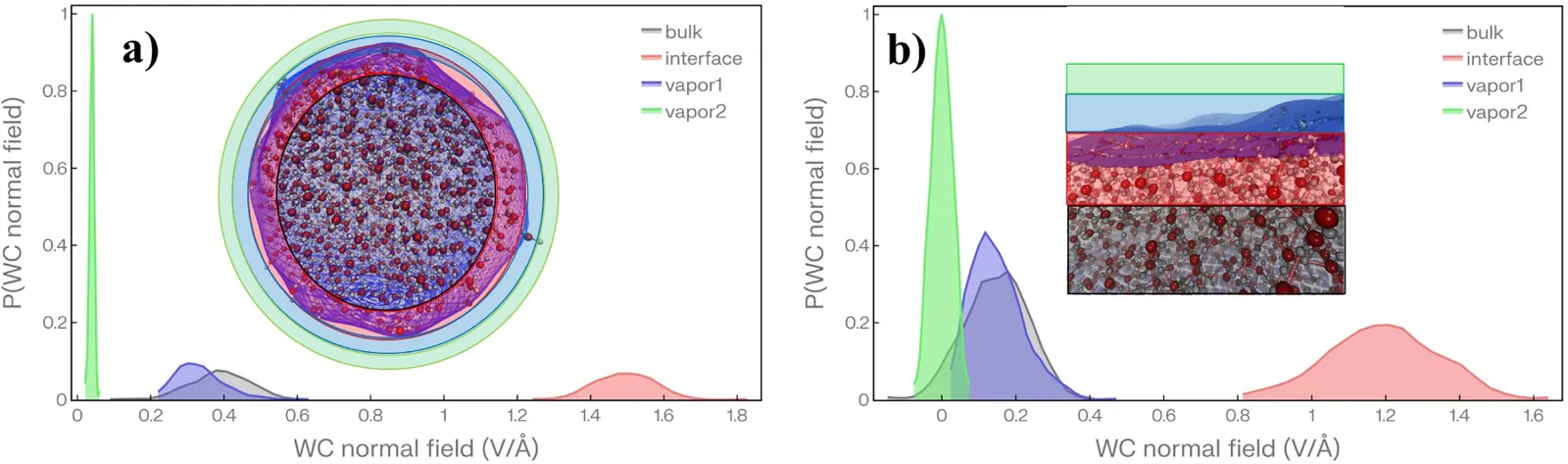
Histograms of the electric field projected along the Willard-Chandler surface normal in different spatial regions. Panel (a) shows the 512 H_2_O nanodroplet geometry, while panel (b) shows the planar slab interface, as also visualized in the respective insets. Distributions correspond to bulk (gray), interface (red), near-vapor (blue), and far-vapor (green) regions. See text for characterization of these regions.

It is important to note that the artificial, non-zero average field observed in the bulk (Figure 4, gray distributions) arises from the cutoff used to define bulk and interfacial regions. This procedure effectively introduces a virtual surface that truncates the hydrogen-bond network at the boundary. As such, the corresponding non-zero average electric field observed in the bulk is not physically meaningful—in a real bulk slab, this field would, in fact, be zero. Although the artificial boundary between the bulk and the interfacial regions also impacts the interfacial field estimate, the fields originating from the interfacial regions are expected to be less affected because these regions already correspond to physically inhomogeneous environments in which hydrogen-bond disruption is intrinsic due to the transition from a condensed phase to vacuum, and will be discussed in the following.

In both droplet (Figure 4a) and slab (Figure 4b) geometries, the largest electric field values are found within the interfacial region. The corresponding distributions peak at approximately 1.2–1.4 V/Å, with strengths slightly higher than those reported in Figures 2–3. This difference arises from the refined definition of the interface adopted here, which excludes lower-field contributions originating from vapor-phase molecules and from regions of vacuum located ≳1 Å above the WC surface, which fall in the vapor1 and vapor2 regions. As anticipated, the combined effects of electron-density spill over, preferential dipolar orientations, asymmetric hydrogen-bonding environments, and enhanced charge fluctuations associated with under-coordinated interfacial molecules give rise to remarkably strong local electrostatic fields within the first molecular layers of the interface.

Moving away from the interface toward the vapor phase, the magnitude of the electric field very rapidly decreases. In the vapor1 region, the typical field values drop to approximately ∼0.3 V/Å, while in the vapor2 region the field vanishes completely within the statistical uncertainty. These results demonstrate that the IEF is localized and decays within only 2.0–2.6 Å from the interface. Beyond the first molecular layers, the electrostatic environment becomes comparable to that of the vacuum.

## Conclusions

In this work, we have provided a fully quantum-mechanical characterization of the electric field at air-water interfaces across planar geometries, nanodroplets of varying curvatures, and under neutral and charged conditions. By combining neural-network-based deep-learning molecular dynamics with *ab initio* electronic-structure resampling, we show that the interfacial electric field (IEF) is an intrinsic feature of aqueous interfaces, consistently oriented outward and governed by the local hydrogen-bond network.

A central result is that both curvature and pH exert only a minor influence on the IEF. Although a systematic linear trend with curvature is observed, its magnitude does not change substantially, and extrapolation to micrometer-sized droplets reveals variations that are orders of magnitude smaller than the field itself. Similarly, while excess charge modulates the field in a predictable manner, its impact becomes noticeable only under extreme pH conditions (*i.e.*, at 13.74 and 0.26). Nonetheless, the changes we observe at these conditions still only involve a marginal variation of ∼10% compared to neutral surfaces. These findings indicate that the experimentally reported reactivity enhancements in microdroplets due to either curvature^33,34^ or charge^73^ cannot be directly attributed to variations in the interfacial electrostatics, but must instead arise from alternative molecular mechanisms. Importantly, the electric field is highly localized, decaying within only 2.0–2.6 Å from the interface. Within this narrow region, the IEF arises from electron density spill over from the liquid to the vacuum and local molecular organization, including dipolar orientation, hydrogen-bond disruption, and electronic polarization, while rapidly vanishing beyond the first molecular layers.

These findings indicate that the electric field is a direct, local manifestation of the underlying molecular and electronic structure. Within this framework, the catalytic properties of aqueous interfaces are more naturally attributed to local chemical effects—including changes in solvation structure and electronic polarization within the first interfacial layers—thereby strongly challenging the common assumption that IEFs are primary driving forces of “on-water” catalysis.

## Supplementary Material



## Notes

Validation of the Deep Potential model for nanodroplets, Willard-Chandler density profiles, hydrogen bonds analyses, size dependence of molecular orientations and geometrical properties, full hydrogen-bond families, interfacial electric field distributions, comparison of the interfacial field with classical SPC/E charges, laterally averaged electrostatic potential, mean inner potential, electrostatic potential gradient, DFT benchmark, grid size benchmark, pairwise structural diversity and dynamical correlations of the selected configurations, assessment of the dipoles behavior in the short-range model, validation of the short-range Deep Potential model against a long-range Deep Potential model, and charge transfer analysis are reported in the Supporting Information file (PDF)
