# Supplementary material for: Interfacial Electric Fields in Water Nanodroplets Are Weakly Dependent on Curvature and pH
Source: J Am Chem Soc. 2026 Oct 8;148(41):44128–37. doi: 10.1021/jacs.6c09391 (PMC13693454; doi:10.1021/jacs.6c09391)
Supplement: Supplementary file 1 [file ja6c09391_si_001.pdf]

Supporting Information

Interfacial Electric Fields in Water Nanodroplets are Weakly Dependent on Curvature and pH

Gabriele Amante,[†,‡] Fortunata Panzera,[†,¶,‡] Pengchao Zhang,[§] Gabriele Centi,[†] Jing Xie,[‖] Ali Hassanali,[⊥] A. Marco Saitta,[¶] and Giuseppe Cassone[*,‡]

†*Department of Chemical, Biological, Pharmaceutical, and Environmental Sciences, University of Messina, Viale F. Stagno d'Alcontres 31, 98166 Messina, Italy*

‡*Institute for Chemical-Physical Processes, National Research Council (CNR-IPCF), V.le F. Stagno d'Alcontres 37, 98158 Messina, Italy*

¶*Laboratoire de physique de L'École normale supérieure de Paris, CNRS, École Normale Supérieure Université Paris Sciences et Lettres, Sorbonne Université, Université de Paris, Paris 75005, France*

§*Center for Combustion Energy, Department of Energy and Power Engineering, and Key Laboratory for Thermal Science and Power Engineering of Ministry of Education, Tsinghua University, 100084 Beijing, China*

‖*Ministry of Education Key Laboratory of Cluster Science, Beijing Key Laboratory of Photoelectronic/Electrophotonic Conversion Materials, School of Chemistry and Chemical Engineering, Beijing Institute of Technology, Beijing 100081 (P. R. China)*

⊥*International Centre for Theoretical Physics (ICTP), Strada Costiera 11, 34151 Trieste, Italy*

E-mail: giuseppe.cassone@cnr.it

Validation of the Deep Potential Model for Nanodroplets

The deep potential (DP) used in the main study is the published model of Ref.[1] To evaluate the sensitivity of its force predictions to model initialization, we trained three additional DP models using the same reference data set, model architecture, loss function, and optimization schedule, but with different initial random seeds. This training design enables the committee analysis below to assess the variation in predicted forces associated with independent parameter initialization.

The models were trained using the DeepPot-SE implementation in DeePMD-kit.[2] The local descriptor used a 6.0 Å neighbor cutoff with smoothing beginning at 0.5 Å, an embedding network with layer sizes [25, 50, 100], and a fitting network with layer sizes [240, 240, 240], tanh activations, and residual connections. Training proceeded for 10 million steps, while the learning rate was reduced from 1×10^{-3} to 1×10^{-8} under the weighted energy-force loss used for the published model.

We have performed a committee-model analysis using four independently trained DP models. For each of 500 configurations from every system, the Cartesian forces on all atoms were evaluated with four independently trained DP models. For each atom i and Cartesian component α, the ensemble mean force and the corresponding overall standard deviation were calculated. The ensemble mean and component wise standard deviation are defined by

$$\sigma_{i,\alpha} = \sqrt{\frac{1}{N_m}\sum_{m=1}^{N_m}\left(F_{i,\alpha}^{(m)} - \overline{F}_{i,\alpha}\right)^2}, \qquad \overline{F}_{i,\alpha} = \frac{1}{N_m}\sum_{m=1}^{N_m} F_{i,\alpha}^{(m)}, \qquad N_m = 4,$$

where each variance is evaluated across the four force predictions. The maximum atomic deviation for each configuration was then defined as $\Delta F_{\max} = \max_{i\in\mathcal{A}} \sigma_i$, where $\mathcal{A}$ denotes the set of all atoms in the configuration. Thus, $\Delta F_{\max}$ provides a conservative measure because each configuration is characterized by the atom displaying the largest disagreement among the four models.

We firstly applied this analysis to the planar slab composed of 256 water molecules already

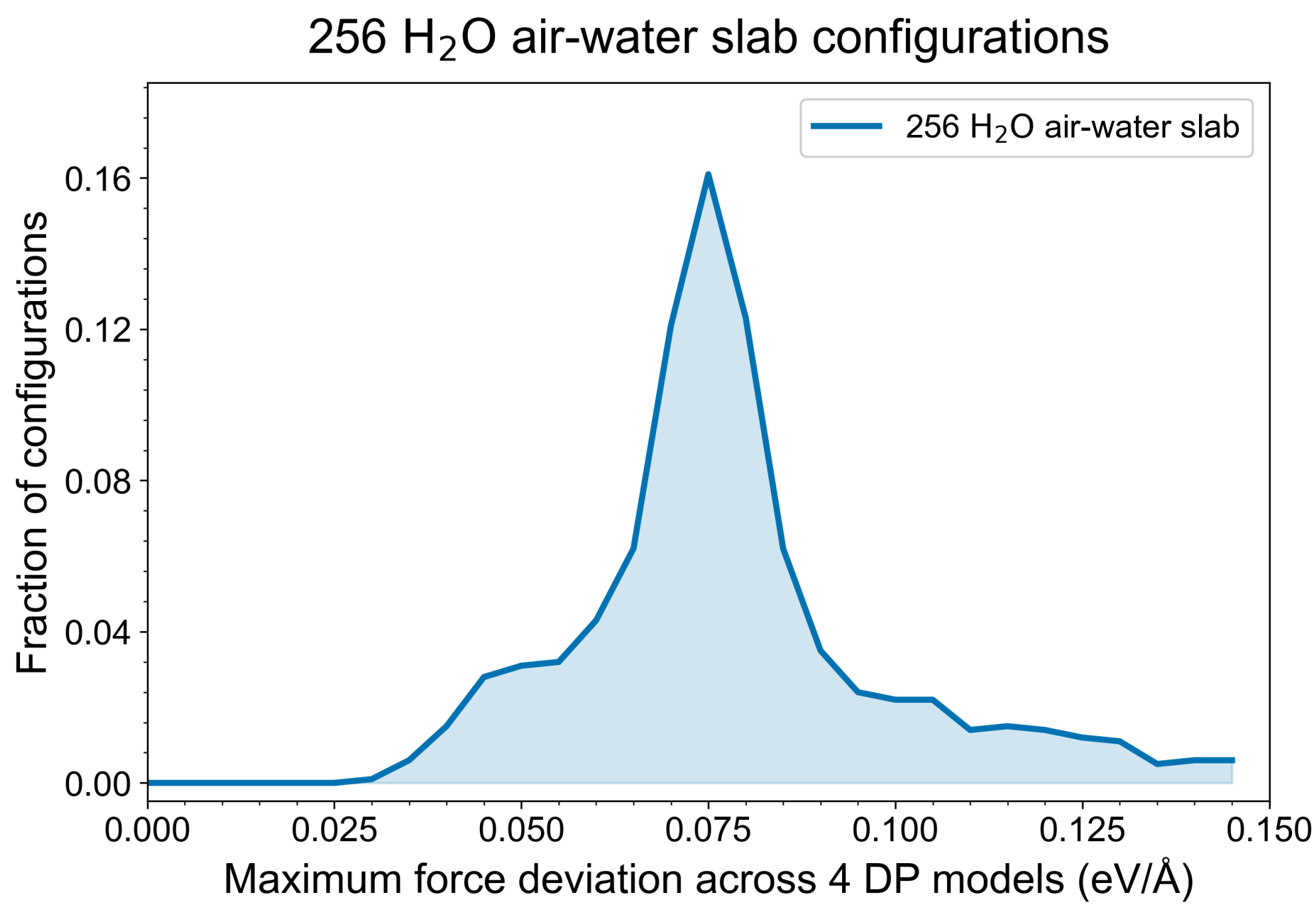

Figure S1: Distribution of the maximum force deviation across four independently trained deep potential models for the flat air-water slab.

validated in Ref.,[1] whose result is reported in Fig. S1. As it is visible, the largest force error is mainly below ∼0.10 eV/Å, suggesting good consistency for the tested slab configurations. We then applied this analysis to neutral droplets containing 256, 512, and 1024 water molecules, as well as to 512-molecule droplets containing either one excess H_3O^+ or one excess OH^- (*i.e.*, 511 H_2O + 1 H_3O^+ and 511 H_2O + 1 OH^-). The respective distributions are reported in Fig. S2. The committee deviations of all nanodroplets are concentrated predominantly between approximately 0.04 and 0.08 eV/Å, with maxima around 0.05-0.07 eV/Å. Only a very small fraction of configurations extends beyond ∼0.10 eV/Å. Importantly, these values fall within the same general range as those of the 256-water planar slab reported in Fig. S1.

The comparison between the 256-water slab (Fig. S1) and the 256-water droplet (Fig. S2, blue curve) is particularly informative because it isolates the change in geometry – from planar to curved – without changing the number of water molecules. No systematic increase in model deviation is observed upon introducing curvature. Similarly, increasing the droplet size from 256 to 1024 water molecules does not produce a progressive shift toward larger

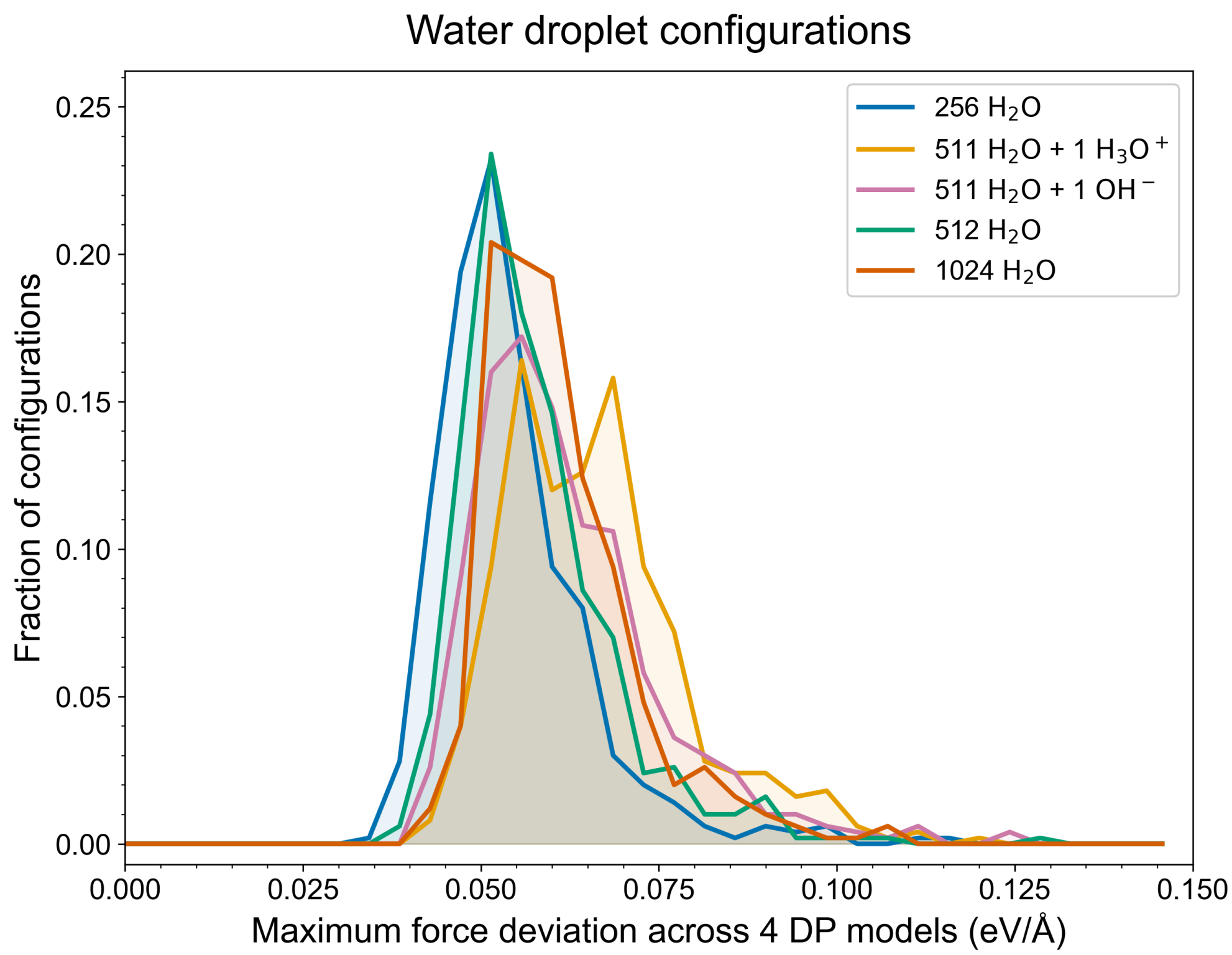

Figure S2: Distributions of the maximum force deviation across four independently trained deep potential models for neutral water nanodroplet systems composed of 256 (blue line), 512 (green line), 1024 (orange line), and of charged droplets containing 511 H_2O molecules and either 1 hydronium (yellow line) or 1 hydroxide (pink line) moiety.

deviations, confirming once again the robustness of the employed neural-network-based potential. The charged droplets show slightly broader distributions than some neutral systems, particularly for the hydronium-containing droplet, but their deviations remain within the range sampled by the neutral droplets and the previously validated slab. Therefore, neither excess hydronium nor hydroxide generates a distinct population of configurations characterized by anomalously large committee disagreement.

The agreement among committee models is an uncertainty indicator rather than an absolute measure of force accuracy: models trained on similar data may, in principle, share a systematic error. We therefore do not present this analysis as a standalone proof of exact equivalence between the DP and the underlying electronic-structure reference. Nevertheless, committee deviation is a sensitive test of whether the new configurations lie outside the configurational domain consistently learned by the independently trained models. The close

overlap between the droplet and slab distributions indicates that curvature, droplet size, and the inclusion of a single water self-ion do not drive the simulations into a substantially less familiar or more uncertain region of configuration space.

With the aim of directly testing and validating energies and forces prediction by the employed DP model, we selected nine representative configurations from the existing trajectories, spanning neutral, hydronium-containing, and hydroxide-containing systems. In particular, three neutral clusters were extracted from a 256-H_2O droplet, three hydronium clusters from a 511-H_2O + H_3O^+ droplet, and three hydroxide clusters from a 511-H_2O + OH^- droplet. Each extracted cluster comprised 32 water-equivalent units: 32 H_2O (96 atoms), H_3O^+ + 31 H_2O (97 atoms), or OH^- + 31 H_2O (95 atoms). Single-point energies and atomic forces were then recalculated at the M06-2X[3] reference level with CP2K. These smaller clusters were required because direct M06-2X calculations for the full production systems are computationally prohibitive.

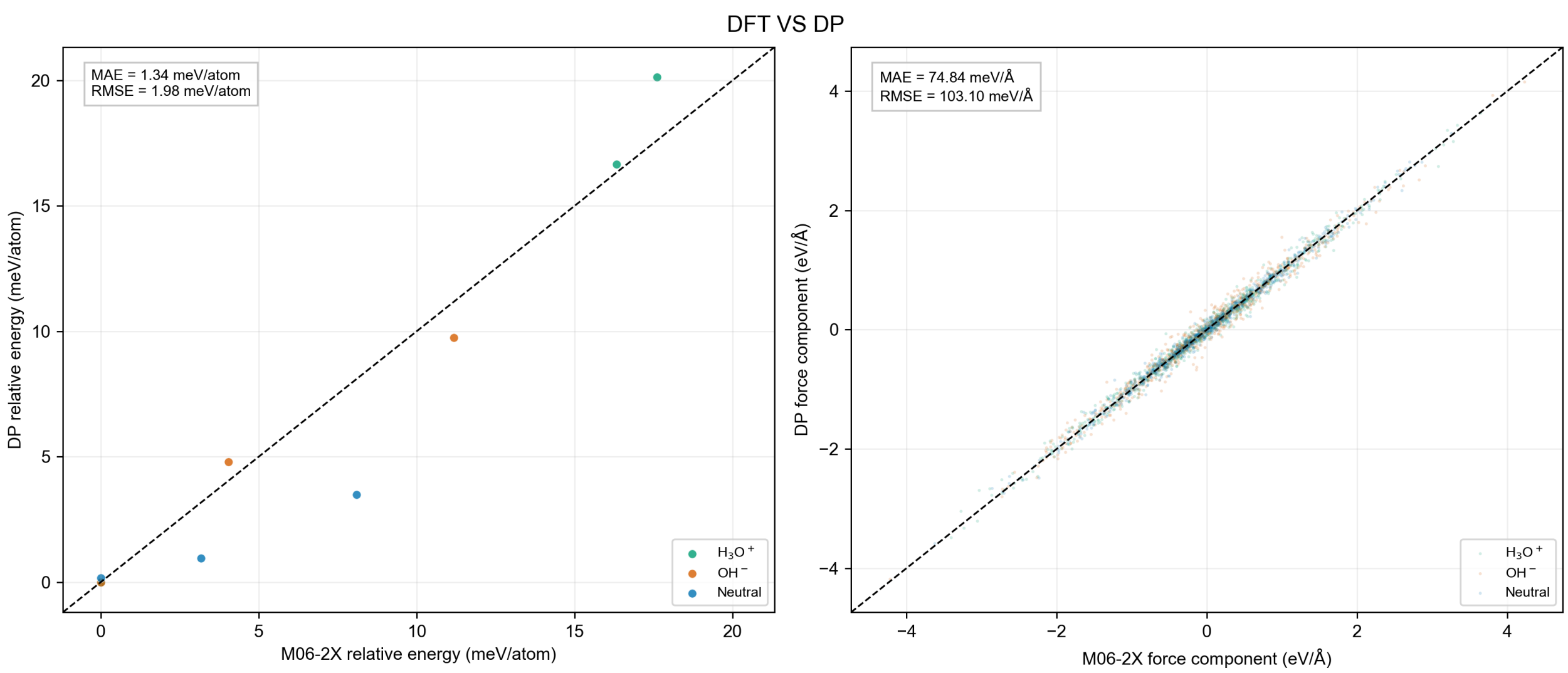

Figure S3: Direct comparison between M06-2X reference data and the short-range Deep Potential model for representative neutral, hydronium-containing, and hydroxide-containing configurations. Left: relative energies per atom. Right: Cartesian force components. The dashed line is y = x.

For each cluster, the three periodic-cell vectors were defined as the corresponding Cartesian span of the cluster plus 24.2 Å, providing a nominal vacuum margin of 12.1 Å on each side. The resulting cell lengths ranged from 32.77 to 35.13 Å. Because the three composi-

tions have different stoichiometries and total charges, the minimum energy was subtracted separately within each composition before comparing relative energies. As shown in Fig. S3, the DP model closely reproduces the M06-2X relative energies and Cartesian force components for the configurations examined. The relative-energy differences have an MAE of 1.34 meV/atom and an RMSE of 1.98 meV/atom, while the force-component differences have an MAE of 74.84 meV/Å and an RMSE of 103.10 meV/Å. The complete numerical results are provided in Table S1.

Table S1: Mean absolute errors (MAEs) and root-mean-square errors (RMSEs) of the energies and Cartesian force components for representative neutral and ionic water clusters as determined from the emplyed DP model and reference M06-2X calculations.

System	Composition	N_{conf}	N_{atoms}	Energy (meV/atom)		Force (meV/Å)	
				MAE	RMSE	MAE	RMSE
Neutral	32 H_2O	3	96	2.336	2.958	57.860	78.936
Hydronium	H_3O^+ + 31 H_2O	3	97	0.952	1.465	77.425	101.991
Hydroxide	OH^- + 31 H_2O	3	95	0.729	0.936	89.346	123.732
All configurations	—	9	—	1.339	1.981	74.835	103.104

Additional Results

As for the flat air-water interface, samples composed of 256 and 512 H_2O molecules were simulated. In addition, seven isolated water nanodroplets composed of 256, 384, 512, 640, 768, 896, and 1024 H_2O molecules were simulated in cubic boxes with edges of 44, 48, 60, 63, 65, 67, and 68 Å, respectively, whereas the respective radii R and the curvature κ are listed in Table S2.

The air-water interface was determined using the Willard-Chandler instantaneous surface definition,[4] which is based on the construction of a local density field obtained by Gaussian smearing of the atomic positions. In this work, the density field was constructed considering only the oxygen atoms of the water molecules, which were chosen as the structural reference to describe the molecular positions. Once the Willard-Chandler surface was determined, the density profile of the system was constructed from the probability of finding an oxygen atom

Table S2: Radius R and curvature κ of the water nanodroplets at $\rho = 1.00\ \mathrm{g\,cm^{-3}}$ investigated in the current study.

N (H_2O)	R (Å)	κ (Å^{-1})
256	12.2	0.0820
384	14.0	0.0714
512	15.4	0.0649
640	16.6	0.0602
768	17.6	0.0568
896	18.6	0.0538
1024	19.3	0.0518

at a given distance from the surface along the local surface normal. The position of the first minimum of this profile was then used as the thickness of the interfacial region. Accordingly, as shown in Fig. S4, the interfacial region includes all molecules located on the vapor side of the surface and extends up to 3.4 Å (for the slab) and 2.6 Å (for the droplet) toward the liquid phase. These corresponds to $d_{WC} = -3.4$ Å (orange line) and $d_{WC} = -2.6$ Å (green line) in Fig. S4, respectively.

To further investigate the origin of the presence of distinct populations of dipoles orientations with respect to the normal to the Willard-Chandler surface in the droplet, resulting in a slightly more well-defined dipole moment direction compared to the flat system, an additional analysis was performed. The density profile reported in Fig. S4 shows the presence of an inflection point at the interface when moving toward the vapor phase. In this region, an inflection point can be identified, located at $d_{WC} = -0.83$ Å for the droplet geometry and $d_{WC} = -1.48$ Å for the flat surface, as marked by the vertical lines L0 of Fig. S5. As reported elsewhere,[5] this feature indicates a change in the structural behavior of water molecules at the interface and justifies the subdivision of the density profile into three distinct regions (L0, L1, and L2), ordered from the vapor side toward the bulk, with L2 exhibiting a bulk-like behavior.

Once the layers were defined, the number of hydrogen bonds per water molecule was computed over the entire trajectories via the standard Luzar-Chandler criterion,[6] distinguishing between intralayer and interlayer contributions (Fig. S5), as a function of the distance

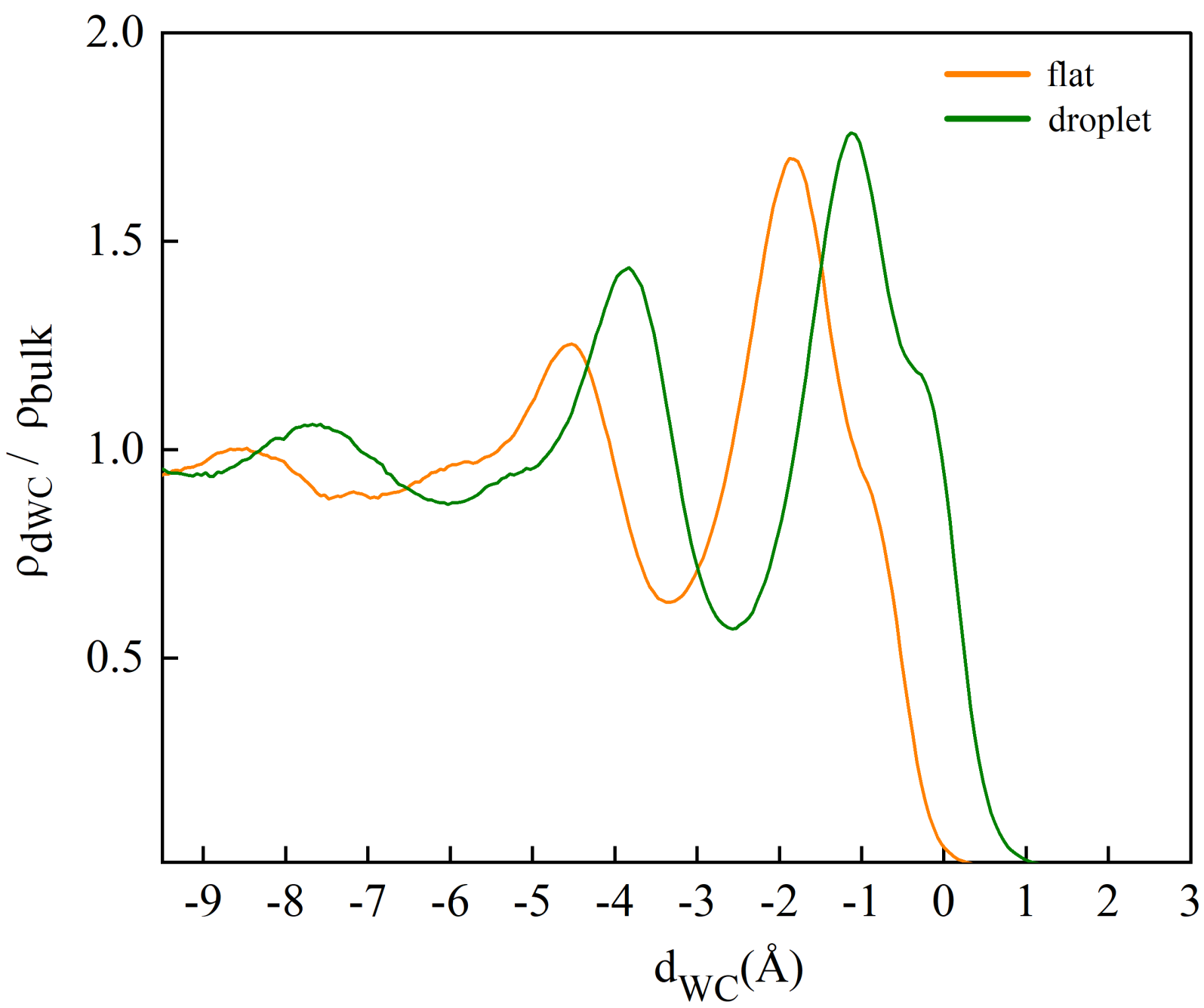

Figure S4: Density profiles sampled as a function of the distance d_{WC} from the Willard-Chandler surface located at $d_{WC} = 0$.

Table S3: Average number of hydrogen bonds per water molecule identified in the L0, L1, and L2 regions.

System	HB_{L0}	HB_{L1}	HB_{L2}
Flat	2.50	3.28	3.51
Droplet	2.40	3.19	3.45

from the Willard-Chandler instantaneous surface. For each hydrogen bond, it was recorded whether the partner molecule belongs to the same layer (intralayer) or to a different one (interlayer), contributing accordingly to the respective counts. Although the average number of hydrogen bonds per water molecule is lower in the droplet than in the flat surface across all layers (Table S3), Fig. S5 clearly shows that, in the droplet, up to a distance of $d_{WC} = -2.6$ Å there is a stronger preference for interlayer hydrogen bonding compared to intralayer bonding, a behavior not so clearly resolved in the flat case, especially in the water phase region of the interface. In fact, for the flat surface, neglecting the discrepancy

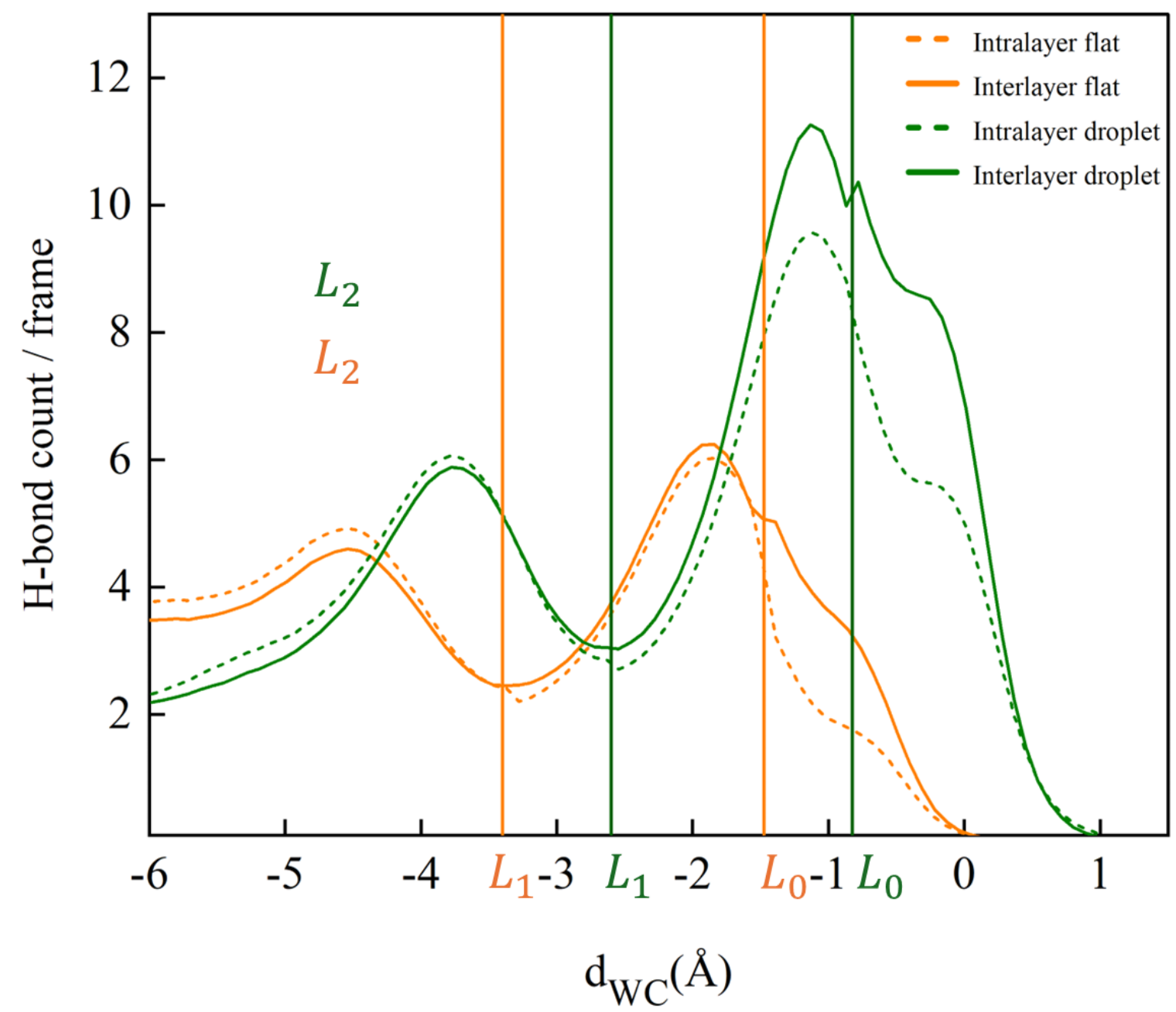

Figure S5: Hydrogen bonds per frame along the depth of the air-water interfaces divided in intralayer and interlayer bonds, for both droplet and flat interface (see legend). Depth thresholds identifying the crossing between different regions are marked as vertical lines.

observed in the outermost region (L0) – which, in addition to having a lower molecular population, exhibits a pronounced vapor-like character, with water molecules being retained at the surface primarily by interactions with the underlying layer – the number of intralayer and interlayer hydrogen bonds becomes nearly equivalent up to the bulk-interface boundary located at $d_{WC} = -3.4$ Å.

In the main text (Figure 1-a), the orientation of the water dipole moments with respect to the Willard-Chandler surface normal have been discussed. In the following Fig. S6, we discuss again those orientations and their direct correlation with the orientations of OH covalent bonds with respect to the same surface normal. Fig. S6 reports the orientational distributions of water dipole moments (α, Fig. S6-a) and OH bonds (β, Fig. S6-b) relative to the instantaneous Willard-Chandler surface normal, for both interfacial and bulk molecules. In the negative $\cos\alpha$ region, interfacial dipoles predominantly orient toward the liquid phase, reflecting asymmetric cohesive interactions. The distribution maximum in Fig.

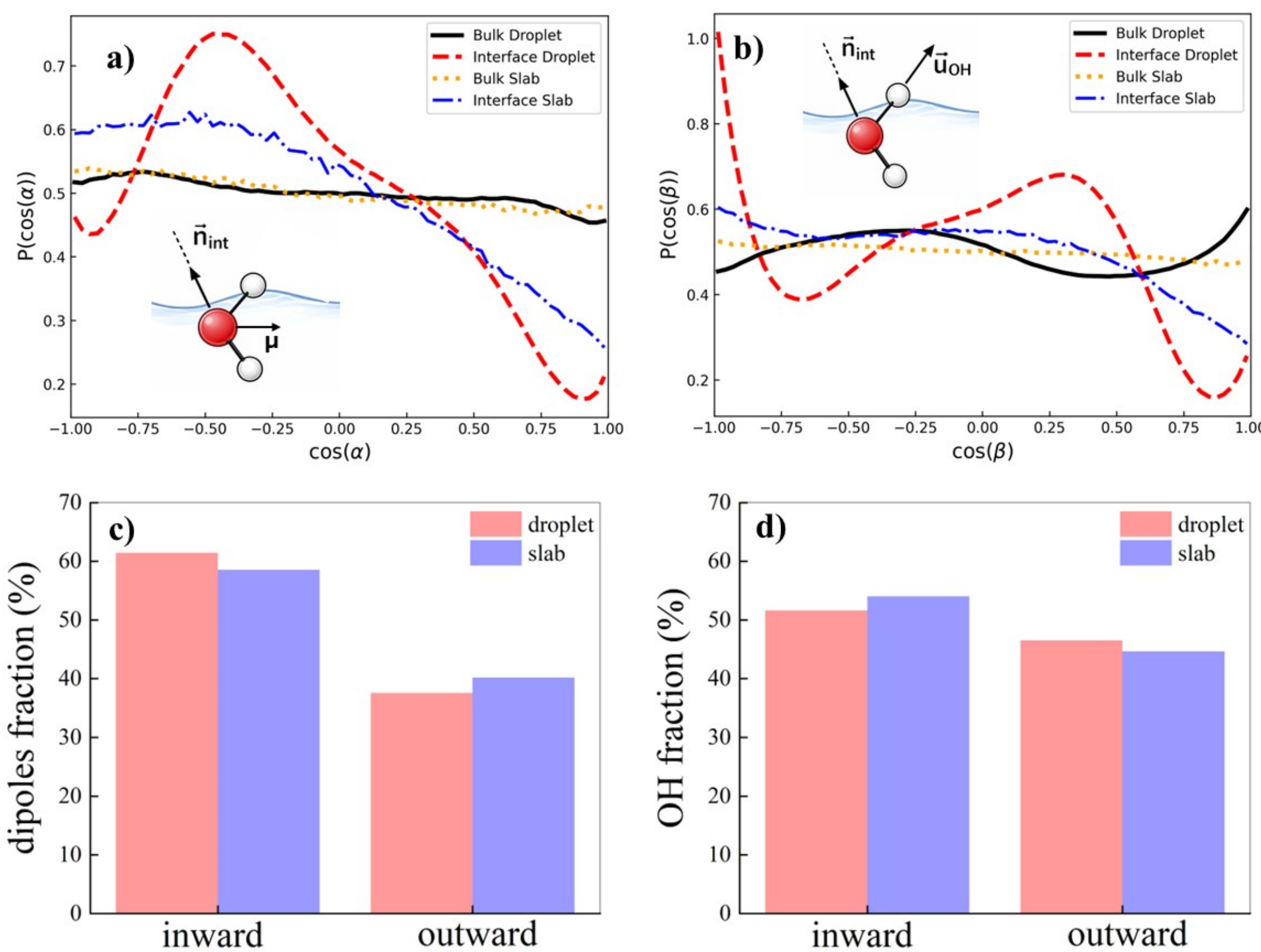

Figure S6: Orientation distributions of molecular dipole moments $P(\cos\alpha)$ (a) and of the covalent O-H bond vectors $P(\cos\beta)$ (b) with respect to the Willard-Chandler surface normal determined in the bulk and at the interfaces of a nanodroplet and a flat slab system composed of 512 H_2O molecules (see legend). In the respective insets, involved vectors are illustrated. (c) Probability of finding inward ($\cos\alpha < 0$) and outward ($\cos\alpha \geq 0$) dipole moment vectors. (d) Probability of finding inward ($\cos\beta < 0$) and outward ($\cos\beta \geq 0$) OH covalent bond vectors.

S6-a at $\cos\alpha \sim -0.45$ corresponds to configurations that favor optimal hydrogen-bonding arrangements. Consistently, the OH orientations (Fig. S6-b) exhibit a correlated pattern: one OH bond points toward the bulk ($\cos\beta \sim -1$), while the other is directed toward the vapor phase ($\cos\beta \sim 0.35$), in agreement with the intrinsic H_2O bond angle.

In the positive $\cos\alpha$ region, associated with dipoles pointing toward the vapor phase, the slab distribution decreases monotonically, whereas the droplet exhibits a noticeable shoulder around $\cos\alpha \sim 0.25$. This feature originates from configurations in which one OH bond remains oriented toward the liquid, thereby stabilizing partially outward-pointing

dipoles. Indeed, inspection of the OH bond orientations in Fig. S6-b reveals a corresponding shoulder at $\cos\beta \sim -0.25$. Therefore, a partial dipole alignment toward the vapor phase ($\alpha \sim 75°$) is stabilized by one OH bond of the same molecule pointing toward the liquid interior ($\beta \sim 105°$). Overall, the air-water interface of curved nanodroplets exhibits a more heterogeneous distribution of molecular orientations than that of flat interfaces, reflecting the larger number of hydrogen bonds formed by water molecules in the planar slab, as discussed above. Nevertheless, the fractions of outward- and inward-oriented molecules remain nearly identical (Figs. S6-c,d), with an excess of dipoles pointing toward the bulk (Fig. S6-c).

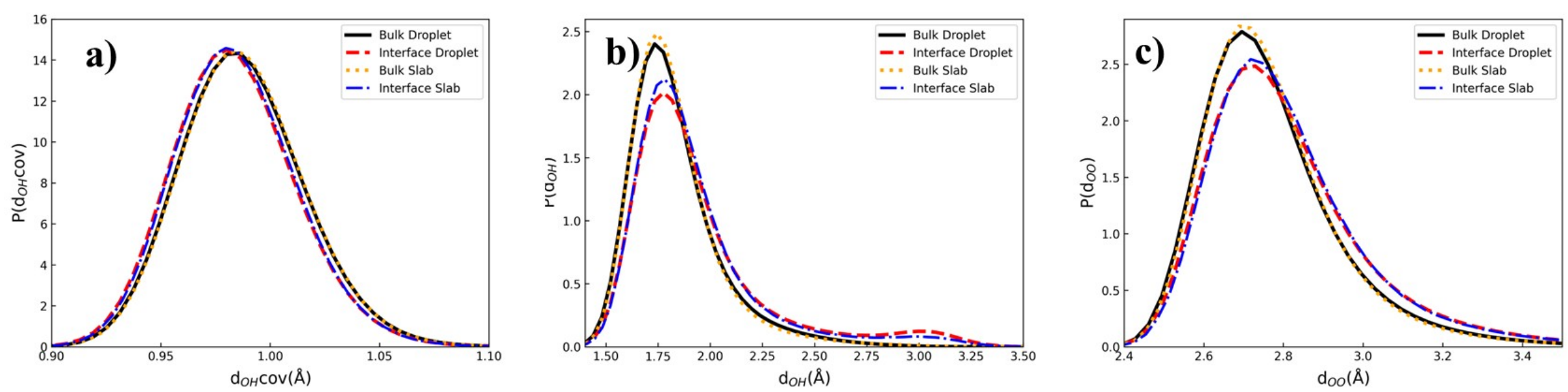

Figure S7: Distributions of the intra- (a), intermolecular O-H (b), and O-O (c) distances in the bulk and at the air-water interfaces of a 512 molecules nanodroplet and flat slab systems (see legends).

Fig. S7 displays the intra- and intermolecular O-H distances in droplet and slab systems, comparing bulk and interfacial regions. In particular, Fig. S7-a reports the covalent O-H bond distances, Fig. S7-b the intermolecular O-H distance, and Fig. S7-c the intermolecular O-O distance. As shown in Fig. S7-a, due to the reduced coordination number associated with the asymmetric local environment, interfacial water molecules are featured by slightly shorter O-H bond lengths compared to the bulk, both in the droplet (dashed red line) and in the planar slab (dash-dotted blue line). As reported in Fig. S7-b, the distance between each oxygen atom and its nearest non-covalently bonded hydrogen atom exhibits a shift toward larger distances in the interfacial region relative to the bulk. Such a behavior is in line with the Maximally Localized Wannier Functions (MLWF) center analysis reported in Figure 1b of the main text, which indicates that interfacial molecules, being less involved in hydrogen-bonding interactions, exhibit lone pairs more localized toward the oxygen atom. Moreover,

compared to the bulk distribution, the interfacial region shows the emergence of a weak additional population around 3 Å, which can be attributed to hydrogen atoms belonging to dangling OH groups oriented toward the vapor phase. Furthermore, the intensity of this band is slightly higher in the droplet, indicating a larger population of dangling OH in the curved droplet than in the planar slab, in line with recent classical molecular dynamics simulations.[7] Within the same lines, also the O-O distance distributions exhibit the trend discussed for the O-H intermolecular distances, although no additional bands are detected at larger distances. This suggests that the extra band observed in Fig. S7-b originates from protons of dangling OH groups at the interface, while the other hydrogen atom of the same molecule directly participates in hydrogen bonding.

The panels of Fig. S8 compare the distributions of the three distances analyzed in Fig. S7 for two droplet systems containing 512 and 1024 water molecules, both in the bulk and in the interfacial regions. All previous considerations hold. In addition, it turns out that the two differently-sized droplets exhibit indistinguishable behavior. This suggests that the droplets size at the nanometric scale does not significantly affect structural properties of the bulk and of the air-water interface.

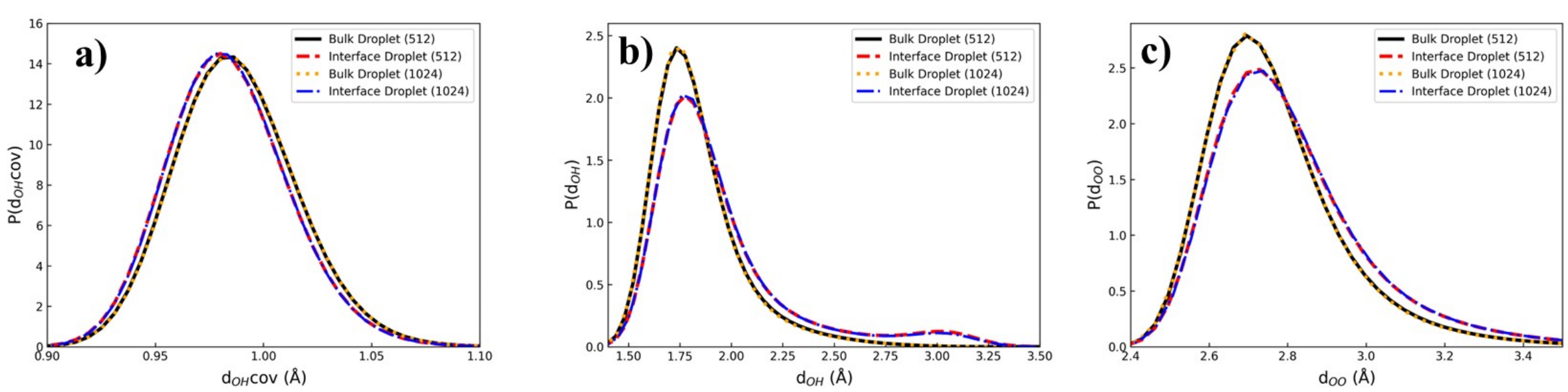

Figure S8: Distributions of the intra- (a), intermolecular O-H (b), and O-O (c) distances in the bulk and at the air-water interfaces of 512 and 1024 molecules nanodroplets (see legends).

To further demonstrate that the droplet size at these scales does not affect the structural properties of the system, we analyzed the distributions of the orientations of molecular dipole moments and covalent O-H bonds relative to the normal to the Willard-Chandler surface, separating the contributions from bulk and interfacial regions. The analysis was

performed for systems containing 512 and 1024 water molecules. As reported in Fig. S9, the comparison between the two systems shows that the resulting distributions are essentially indistinguishable.

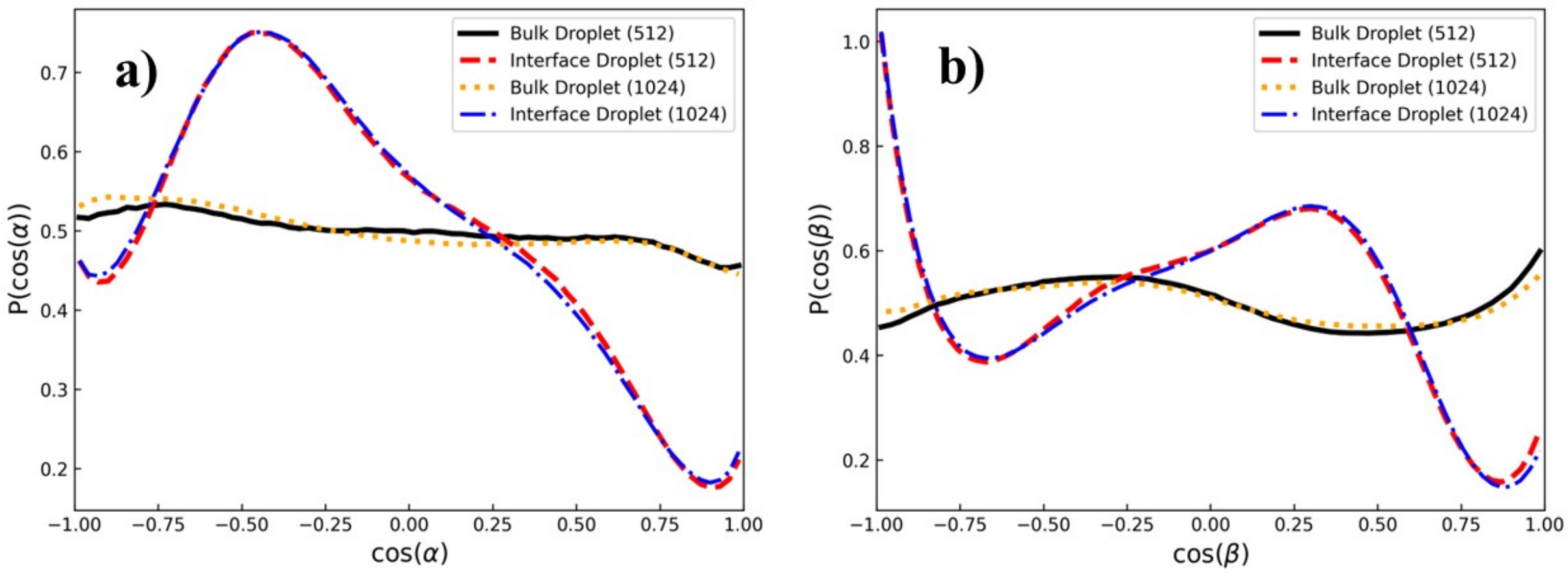

Figure S9: Orientation distributions of molecular dipole moments P($\cos\alpha$) (a) and of the covalent O-H bond vectors P($\cos\beta$) (b) with respect to the Willard-Chandler surface normal determined for nanodroplets composed of 512 and 1024 H_2O molecules (see legends).

Fig. S10 reports the full, unfiltered set of hydrogen-bonded molecular families distributions complementing the selected profiles shown in the main text in Figure 1c,d. Exotic configurations such as 0D-0A, 2D-4A, or configurations in which the total number of hydrogen bonds is greater equal 6 ($HB_{tot} \geq 6$) carry negligible statistical weight at every depth from the Willard-Chandler surface in both the nanodroplet and slab geometries, confirming that their omission from the main discussion in the main text does not affect the overall physical picture.

As detailed in the Methods section of the main text, for each grid point of the electric field, the corresponding normal vector to the intrinsic surface, $\boldsymbol{n}_{\text{int}}$, was computed. The electric field vector $\boldsymbol{E} = (E_x, E_y, E_z)$ was then projected onto the local Willard-Chandler reference frame and the normal component of the electric field was obtained as $E_n = \boldsymbol{E} \cdot \boldsymbol{n}_{\text{int}}$. Finally, the projected field components were accumulated over all grid points belonging to the same spatial region. Using this interfacial field definition, the electric-field distributions

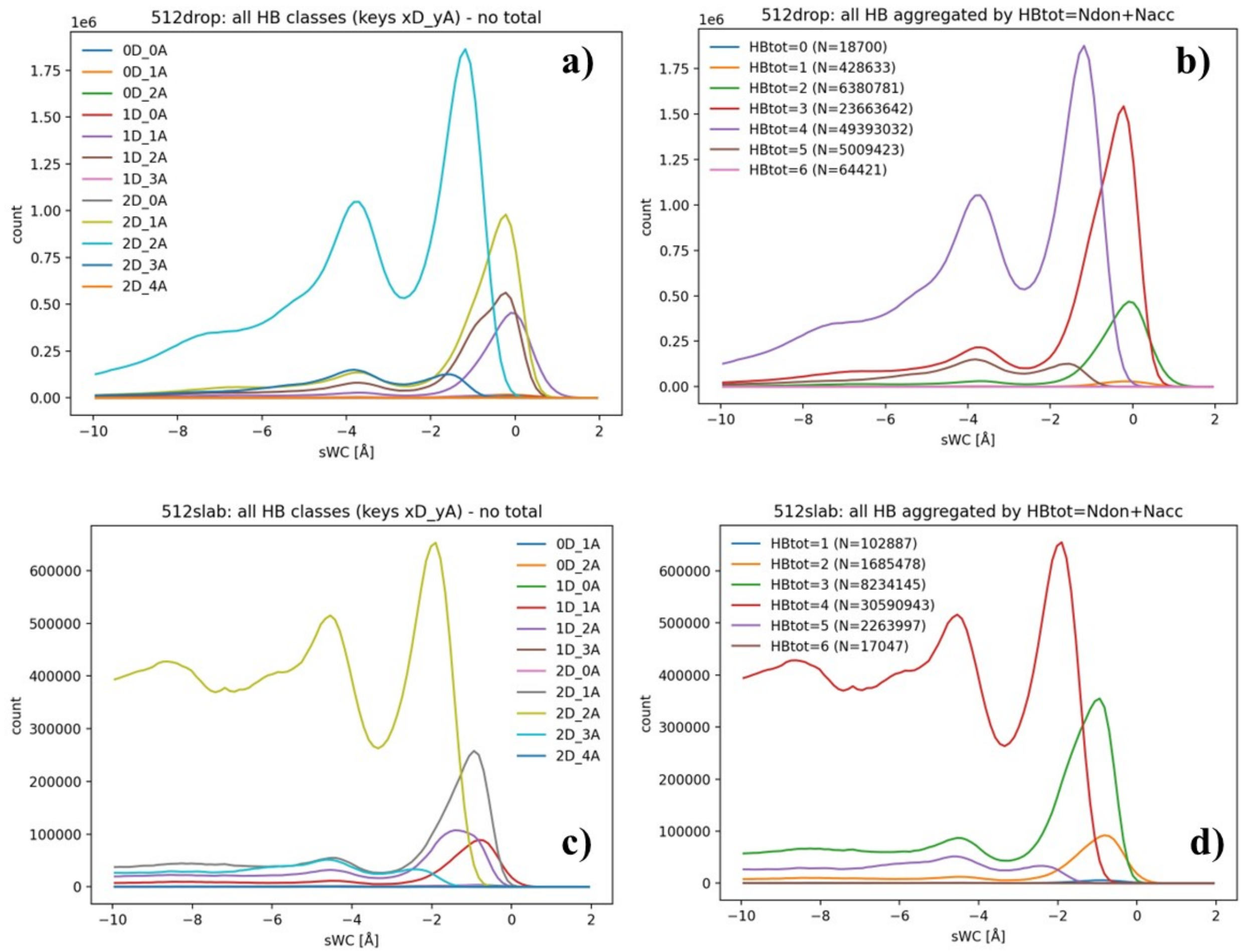

Figure S10: Hydrogen-bond family distributions as a function of the Willard-Chandler distance s_{WC} for the 512 molecule nanodroplet (a, b) and slab (c, d). Panels (a, c) resolve individual xD_yA classes; panels (b, d) aggregate by total coordination $\mathrm{HB}_{\mathrm{tot}} = N_{\mathrm{don}} + N_{\mathrm{acc}}$, with total counts N in the legends.

are Gaussian-like across all systems' sizes investigated in the current work, as shown in Fig. S11-a. It is worth pointing out that a detailed analysis of the corresponding fluctuations – which was reported to be relevant in Lorentzian-like distributions under different interfacial field definitions in Ref.[8] – is limited by the relatively small amount of data points (i.e., one per configuration, which corresponds to 500 data points per system). The indication we get from these data is that no clear dependence on the system's size is observed from our distributions, with exception of the significantly larger fluctuations exhibited by the flat slab system where, on the other hand, the smallest field values were recorded. However, the corresponding variation range is on the order of less than 25% with respect to its mean value.

The latter fluctuation is hence not comparable with those reported by Head-Gordon and co-workers[8] for droplet systems, who show that fluctuations ≈30 times larger than the average interfacial electric field are present and lead to non-Gaussian (i.e., Lorentzian) distributions.

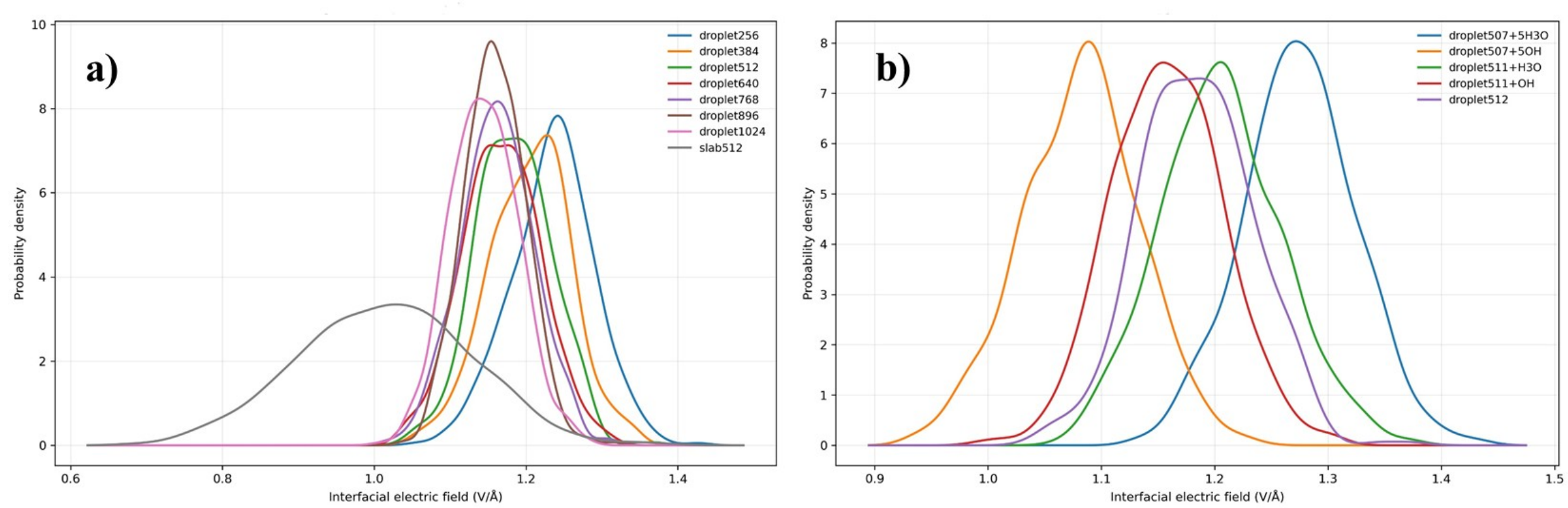

Figure S11: (a) Distributions of the interfacial electric field projected onto the surface normal of the Willard-Chandler instantaneous interface across different nanodroplet sizes and a flat air-water slab (see legend, where the number corresponds to the amount of water molecules). (b) Distributions of the same quantity across differently charged nanodroplets (see legend).

Within this context, it might be interesting to evaluate whether the inclusion of water's counterions impacts the extent of the interfacial electric field fluctuations. As displayed in Fig. S11-b, the broadness of the field distributions is not affected by the inclusion of different amounts of either OH^- or H_3O^+ species. In fact, the full widths at half maximum of the respective distributions are comparable to that corresponding to the neutral case (violet curve). As discussed in the main text of the manuscript (Figure 3), we do observe only a rigid shift of the mean of the distributions, with symmetrical behavior of positively and negatively charged droplets with respect to the neutral reference case. Similarly to the previously discussed neutral case, the associated distributions are Gaussian-like.

To establish a direct comparison with the distributions reported by Head-Gordon and co-workers, we have additionally recomputed the electric field using the definition they report in equation (4) of Ref.[8] but from our quantum electron density for a nanodroplet and a slab system composed of 512 H_2O molecules each. As shown in Fig. S12, this separate

analysis indicates that, when defined in this way, the distribution recovers the same qualitative Lorentzian character reported in that work. Notably, even under this independent definition, the median interfacial electric field of the water nanodroplet remains larger – while still within the same order of magnitude – than that of the planar slab interface (0.89 vs 0.33 V/Å, respectively). This observation further supports the conclusion drawn from Figure 2 of the main manuscript that curved interfaces exhibit stronger interfacial electric fields than their planar counterparts. The difference in distribution shape between Fig. S11 and Fig. S12 should not be attributed solely to the definition of the electric field. The level of sampling is also different: our Willard-Chandler analysis constructs a distribution from one spatially averaged interfacial value per configuration, whereas the Head-Gordon analysis samples local grid-point values. Spatial self-averaging necessarily suppresses local fluctuations and can promote a narrower, more Gaussian-like distribution. Future work from our group will focus on understanding the microscopic origins of the statistical distributions associated with different constructions of the electric fields.

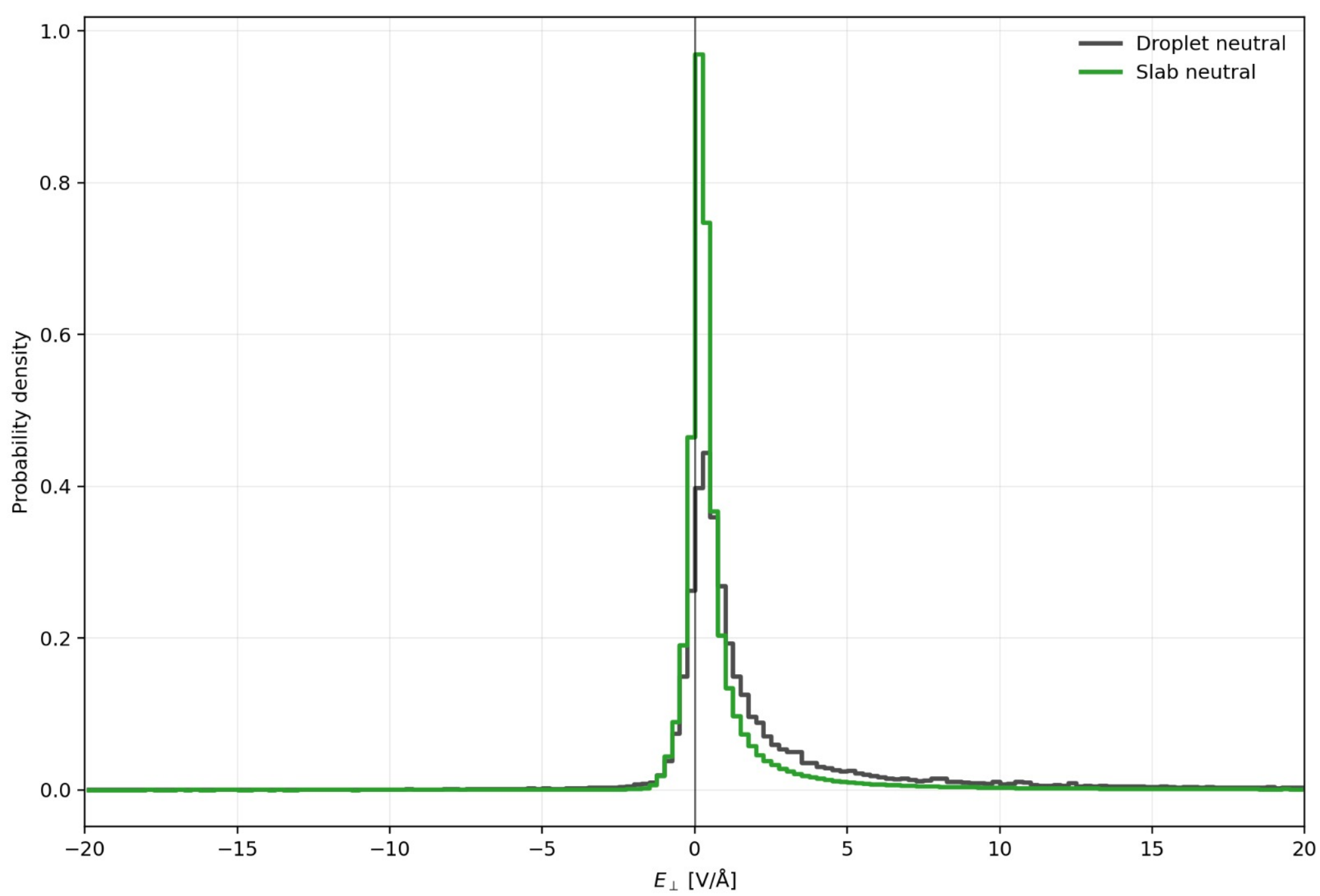

Figure S12: Distribution of the interfacial electric field defined as in equation (4) of Ref.[8] for a water nanodroplet (black line) and a flat air-water slab (green line).

It is instructive to monitor how the field distributions change when using force-field charges. Usage of classical charges is often useful because of the associated reduction in computational cost with respect to the determination of the DFT electron density. However, such a reduction of information on the spatial modulation of the charge in condensed matter is not free from impacting the determination of the electric field. As an exemplary test, we have determined the electric field distribution by dressing 5000 nuclear configurations of the 512 H_2O flat air-water slab from our DPMD simulations with SPC/E charges (i.e., O= $-0.8476\ e^-$ and H=$+0.4238\ e^-$) and compared it with the same quantity determined via Poisson's equation from the corresponding quantum electron density, the latter calculated over 500 configurations only. The results are reported in Fig. S13. Interestingly, the mean electric field computed from classical point charges is only ≈0.1 V/Å, whereas the average field obtained by projecting the DFT-derived electric field onto the Willard-Chandler surface normal is approximately one order of magnitude larger, reaching ≈1.0 V/Å. Notably, as we will show below, the latter is in much closer agreement with the field strength inferred from the experimentally relevant laterally averaged electrostatic potential (≈1-2 V/Å).

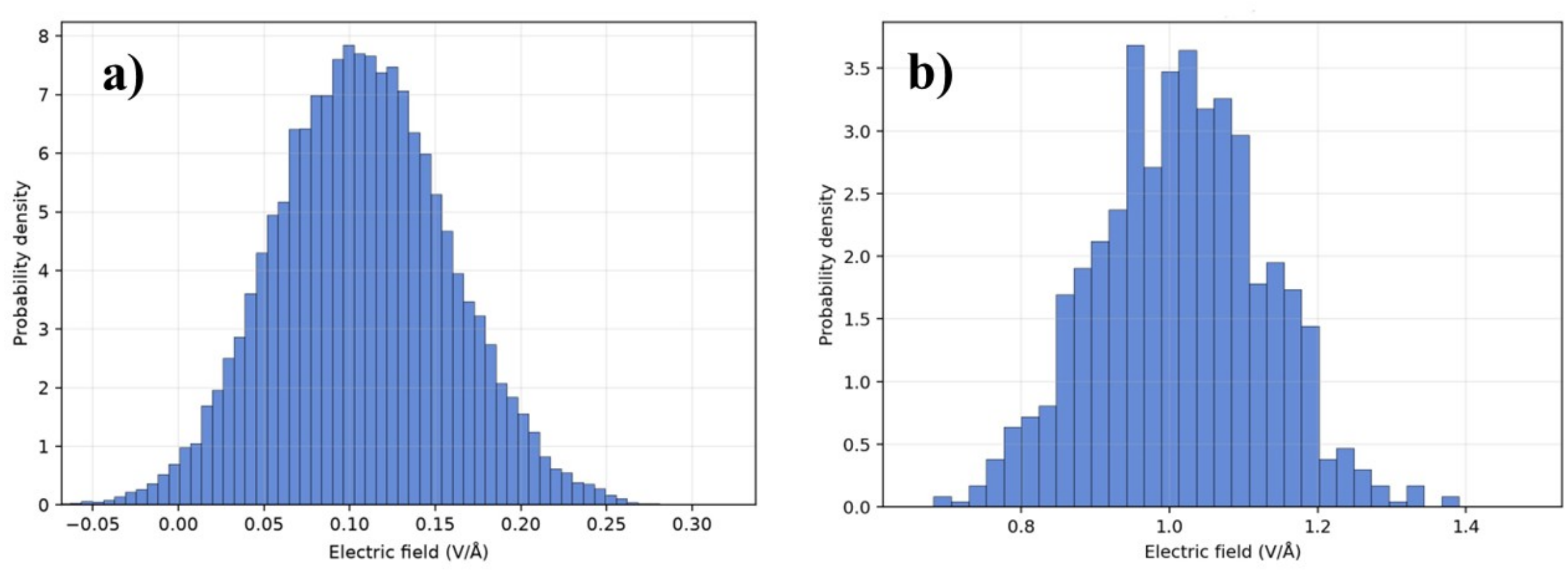

Figure S13: Histograms of the interfacial electric field projected on the Willard-Chandler surface normal of the flat air-water slab nuclear configurations extracted from our DPMD trajectory and determined by employing SPC/E charges (a) and the DFT electron density (b) as field sources.

To relate the local Willard-Chandler projected fields reported in the current work to experimentally measurable electrostatic observables, such as the electrostatic potential and

the corresponding mean inner potential (MIP),[9] we have re-sampled 100 randomly chosen configurations of the flat air-water slab system composed of 512 H_2O molecules to extract the laterally averaged electrostatic potential profile V(z). From the latter we have determined the corresponding MIP, displayed in Fig. S14, whose average value falls at 5.18 V at the PBE+D3[10–12] level. Such value is larger by ≈ 16% than the experimentally measured one (i.e., 4.48 V).[9] As discussed in Ref.,[9] a pseudopotential DFT electrostatic potential may not

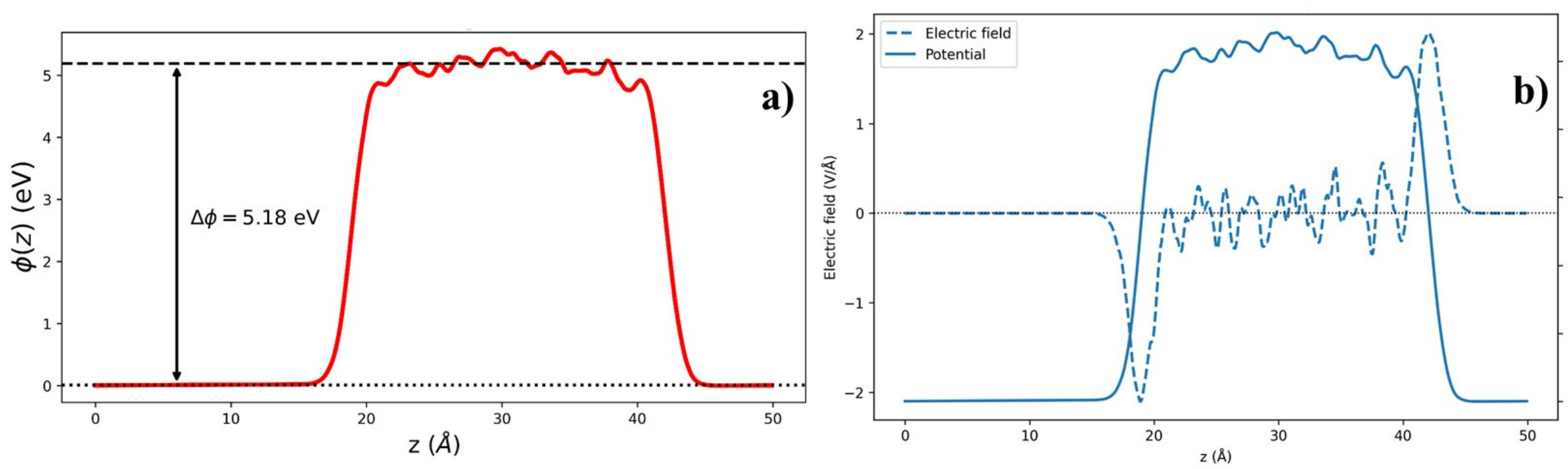

Figure S14: (a) Laterally averaged total electrostatic potential – determined at the PBE+D3 theory level – averaged over 100 randomly chosen configurations extracted from our DPMD simulation of a flat air-water slab composed of 512 water molecules. (b) The same electrostatic potential (solid blue line) plotted together with its negative derivative corresponding to the electric field (dashed blue line).

be directly comparable to the experimental value. In fact, the MIP is highly sensitive to the treatment of the nuclear cores and the pseudopotentials of Goedecker-Teter-Hutter (GTH)[13] quality certainly impact the determined MIP value. Furthermore, another factor might be due to the well-known self-interaction error of GGA-quality exchange-correlation functionals, even though nuclear configurations stem from a machine-learning potential trained on DeePKS/M06-2X data going beyond the GGA approximation. As for the latter point, however, the impact of the electronic structure quality appears to be quite limited on the evaluation of the (strong) interfacial electric fields projected on the Willard-Chandler surface normal (see below). On top of this, it is instructive to determine the electric field profile as the negative derivative of the laterally average electrostatic potential (i.e., E(z) = -dV(z)/dz), a quantity plotted in Fig. S14-b (blue dashed line). Notably, the direction of the field is consistent with that determined from our method (i.e., outward from the liquid and

toward the vacuum) and, importantly, the peak of the electric field profile in the interfacial region reaches a value of ≈ 2 V/Å, in line with average interfacial electric field strengths projected onto the Willard-Chandler surface normal we discuss in our work (≈ 1 V/Å) and with previous estimates by Kathmann and co-workers.[14] All these differently-derived results – which include three different electric field definitions and an independent re-sampling with classical SPC/E charges directly linking the current work to previously published works – explicitly demonstrate that different constructions of the field probe different field magnitudes, but not different field signs (*i.e.*, it is always outward), while also showing that the principal comparative trends identified in this study are not tied exclusively to a single statistical representation and naturally reconciles with previous works.

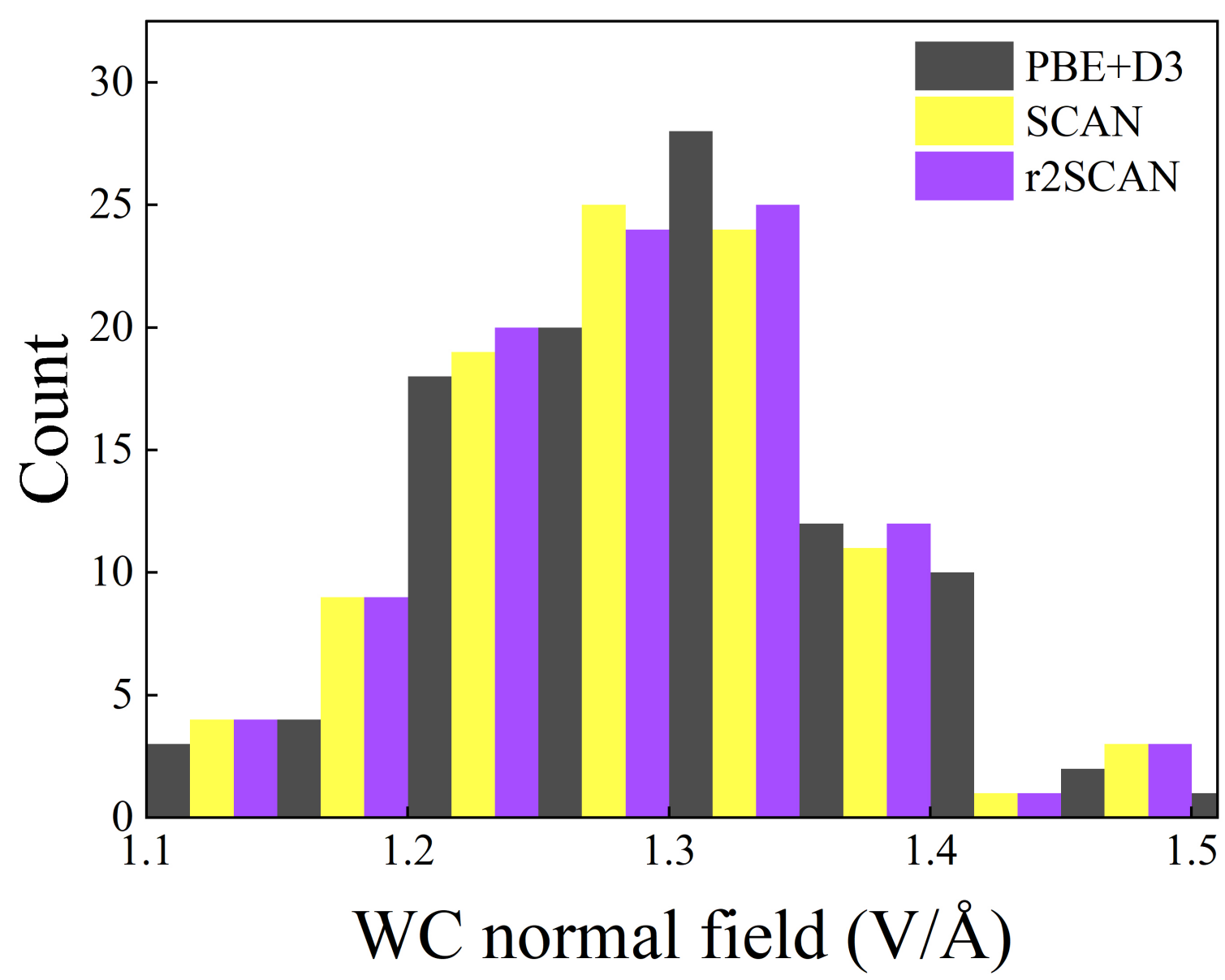

Figure S15: Histograms of the interfacial electric field projected onto the Willard-Chandler surface normal for a relatively small 128 molecule nanodroplet computed with PBE+D3, SCAN, and r^2SCAN exchange-correlation functionals.

Fig. S15 compares the distribution of the Willard-Chandler normal electric field obtained with PBE+D3,[10–12] SCAN,[15] and r^2SCAN[16] for the 128 molecule nanodroplet. The three functionals yield nearly overlapping histograms, with differences within statistical noise. This agreement justifies the adoption of the PBE+D3 functional for the larger systems investi-

gated in the current work, where the meta-GGA functionals SCAN and r^{2}SCAN cannot be employed due to the sizably larger RAM requirements.

We have also benchmarked the impact of the grid size of the mesh where the electric field is printed from self-consistent field calculations. In particular, we compared the average interfacial electric field projected on the Willard-Chandler surface normal for selected nuclear configurations extracted from our DPMD simulations of the flat air-water slab containing 512 H_2O molecules and determined over a very dense uniform grid mesh with 0.08 Å spacing and a mesh twice larger (i.e., 0.16 Å). As displayed in Table S4, the largest relative change is about 3.9%, while most rows differ by less than 2%, indicating that at these regimes of data points density only minor effects on the computed electric field are observed.

Table S4: Comparison between the interfacial electric field projected on the Willard-Chandler surface normal computed with grid meshes with spacing of 0.08 Å (first column) and 0.16 Å (second column). Third column reports the difference while the last column reports the relative percentage variation.

0.08 Å	0.16 Å	Difference	Δ (%)
1.005645	1.022159	−0.016514	+1.642
1.262532	1.220801	+0.041731	−3.305
1.001107	1.001059	+0.000048	−0.005
1.119786	1.077555	+0.042231	−3.771
1.471955	1.470609	+0.001346	−0.091
1.116349	1.078955	+0.037394	−3.350
1.234327	1.263693	−0.029366	+2.379
1.092316	1.085383	+0.006933	−0.635
1.202220	1.228960	−0.026740	+2.224
1.145543	1.111909	+0.033634	−2.936
1.361875	1.356670	+0.005205	−0.382
1.283963	1.266045	+0.017918	−1.396
1.039575	1.045749	−0.006174	+0.594
1.120029	1.120029	0.000000	0.000

Ascertained the quality of the DFT protocol used, it is important to clarify whether the 500 configurations randomly extracted from each system reflect sufficiently distinct atomic arrangements. As an additional and orthogonal check on redundancy, we evaluated all C(500,2) = 124750 pairwise Euclidean distances in the non-periodic DeepMD descriptor space for the neutral air-water slab composed of 512 water molecules, the neutral nanodroplet

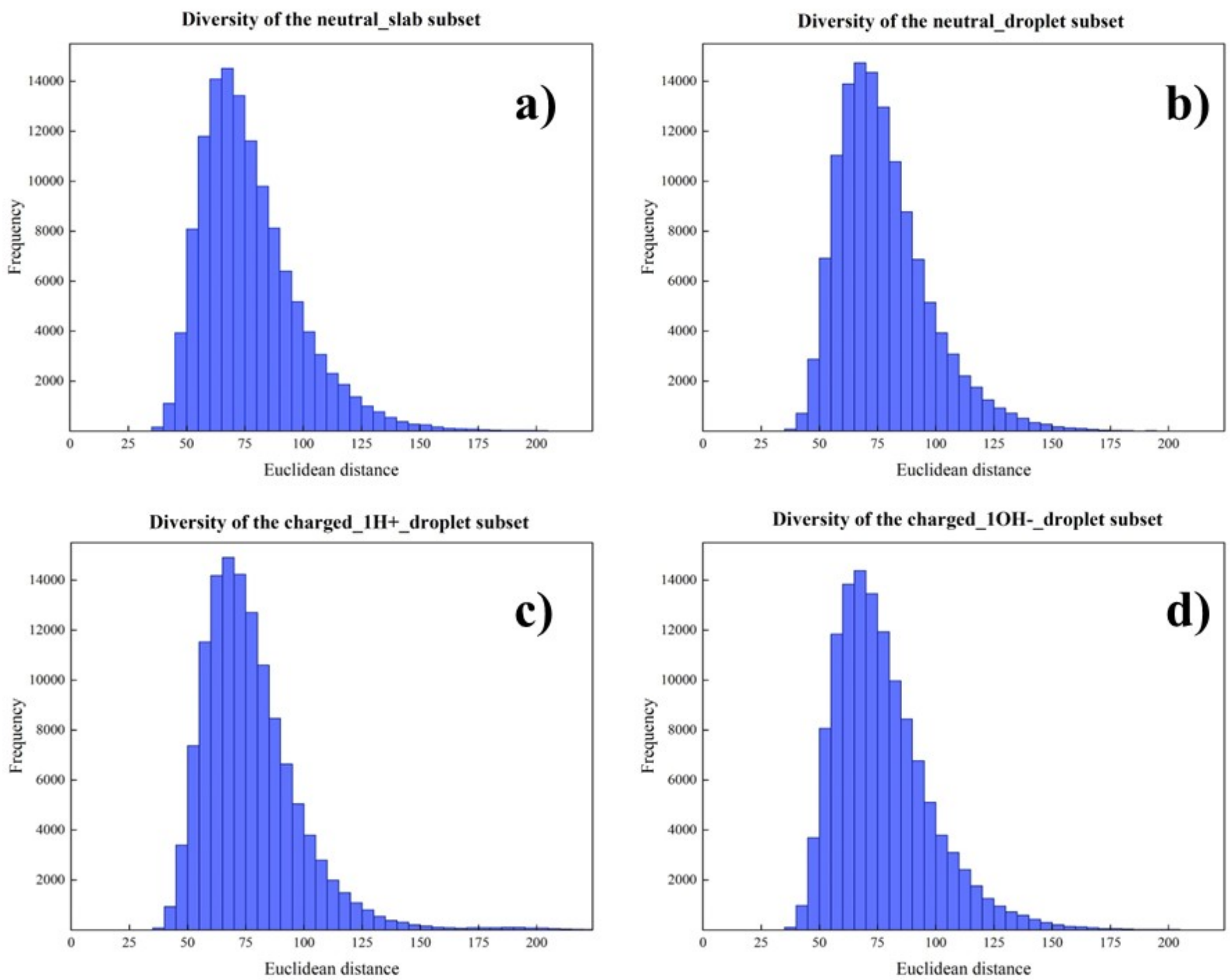

Figure S16: Pairwise structural diversity among the 500 re-sampled configurations for (a) a neutral air-water flat slab (without periodic boundary conditions), (b) a neutral nanodroplet, (c) a hydronium-containing nanodroplet, and (d) a hydroxide-containing nanodroplet. The histograms contain all 124750 pairwise Euclidean distances in the non-periodic DeepMD descriptor space for each dataset.

of the same size, the hydronium-containing, and hydroxide-containing datasets. Fig. S16 shows broad distributions well separated from zero in all cases, demonstrating that the DFT re-sampling set spans substantial geometric diversity and is not dominated by duplicate, sizably overlapping, or nearly identical structures.

On the other hand, that descriptor-space diversity does not report on dynamical correlations between configurations and it only addresses the narrower question of whether the selected electronic-structure calculations sample a geometrically redundant set. To address the issue of correlations between the randomly selected configurations, we report in Fig. S17, the pairwise temporal distance between the 500 selected configurations compared with the integrated autocorrelation characteristic time τ_{ACF} of the DeepMD latent feature vectors across the main systems used in this work. In particular, we observe that τ_{ACF} falls between

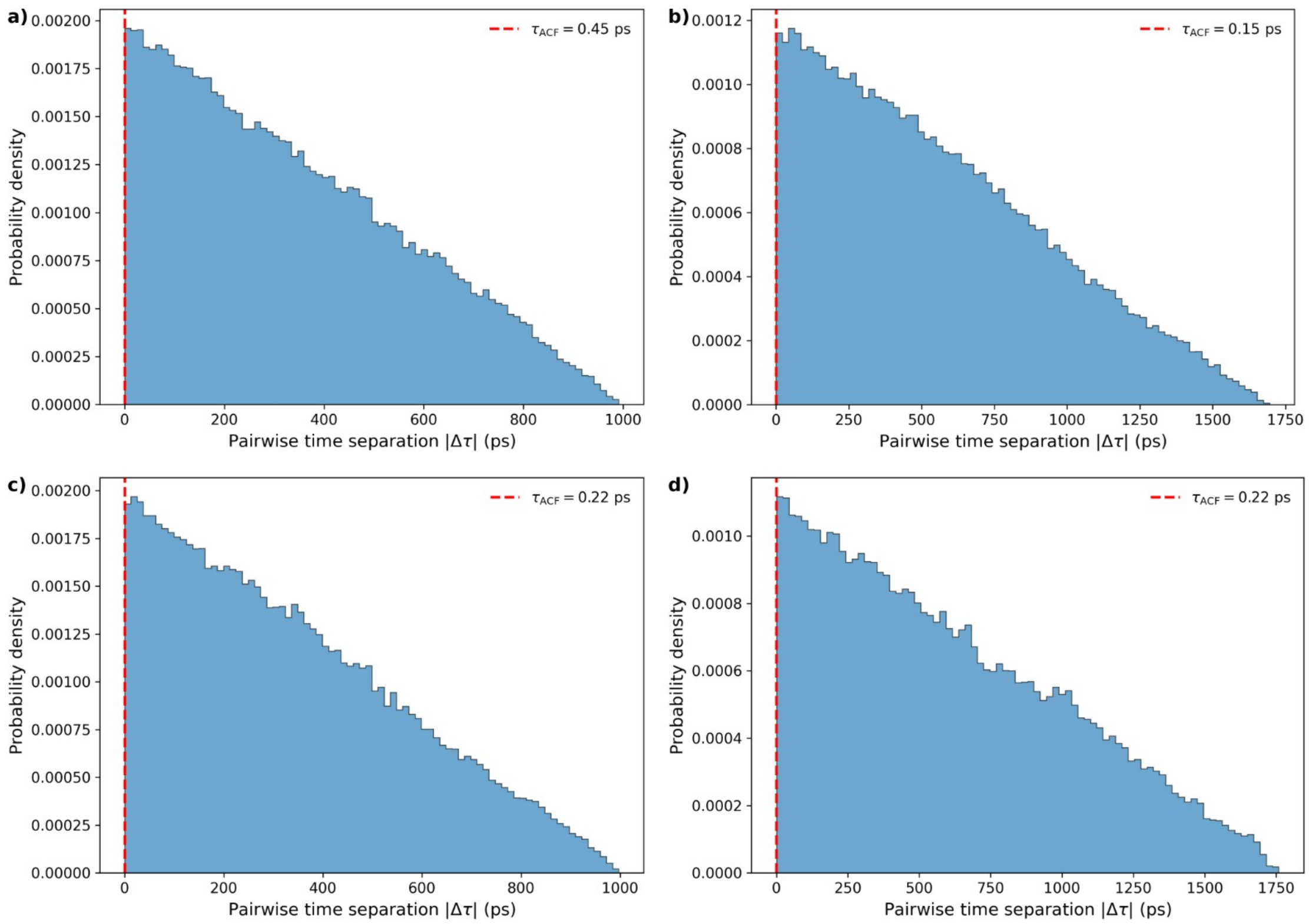

Figure S17: Pairwise temporal distance between the 500 selected configurations across the air-water slab (a), the neutral isolated 512 H2O nanodroplet (b), the hydronium-containing nanodroplet (c), and the hydroxide-containing nanodroplet (d) subsets compared with the integrated autocorrelation characteristic time τ_{ACF} of the DeepMD latent feature vectors (red dashed vertical line). In all cases, more than 99.9% of the selected configurations are well above τ_{ACF}.

$\sim 0.15 - 0.45$ ps, already indicating a decorrelation process sizably faster than the sampled 1000 ps (1 ns) simulation time. Furthermore, as can be seen from Fig. S17, in all systems, more than 99.9% of the the pairwise temporal distance between all sampled configurations falls well above the corresponding autocorrelation time τ_{ACF}, a circumstance further highlighting the that the selected configurations subsequently used for *ab initio* re-sampling are not correlated in structure (Fig. S16) and in time (Fig. S17).

Assessing the Dipoles Behavior in a Short-Range Model

The use of a short-range deep-learning potential at a liquid-vapor interface requires explicit validation. In particular, the absence of long-range electrostatics may, in some systems, produce an unphysical orientational response[17] extending far from the interface and anomalously large collective dipole fluctuations, potentially affecting electronic properties subsequently calculated for configurations generated with the potential. We have therefore performed an additional and independent validation of the molecular orientational structure predicted by our short-range deep potential molecular dynamics (DPMD) model. Specifically, we carried out a simulation of a neutral nanodroplet containing 512 H_2O molecules using MB-pol(2023)[18] and analyzed the resulting configurations using exactly the same definition of the instantaneous interface, spatial partitioning, and dipole-orientation angle adopted for the DPMD trajectories. MB-pol(2023) provides a particularly stringent reference because it is a data-driven many-body polarizable potential based on high-level coupled-cluster calculations and explicitly accounts for longer-range electrostatics and many-body polarization. The comparison is reported in Fig. S18. In the bulk-like region of the droplet, both DPMD and MB-pol(2023) yield a nearly uniform distribution of $\cos\alpha$, as expected for isotropically oriented molecular dipoles. Importantly, neither model displays a persistent orientational bias extending into the droplet interior, as expected for a pathological short-range machine-learning interatomic potential. At the interface, the two models also predict the same qualitative structural features: a principal maximum for negative $\cos\alpha$ values, a weaker shoulder at moderately positive $\cos\alpha$ values, and minima near $\cos\alpha \approx 1$. Although the two distributions do not coincide quantitatively at every value of $\cos\alpha$ at the interface, their overall agreement shows that the interfacial orientational motifs discussed in the manuscript are independently reproduced by a model that explicitly includes longer-range electrostatics and polarization.

We emphasize that the MB-pol(2023) comparison concerns a neutral water droplet and therefore primarily validates the solvent orientational structure rather than the ion-specific

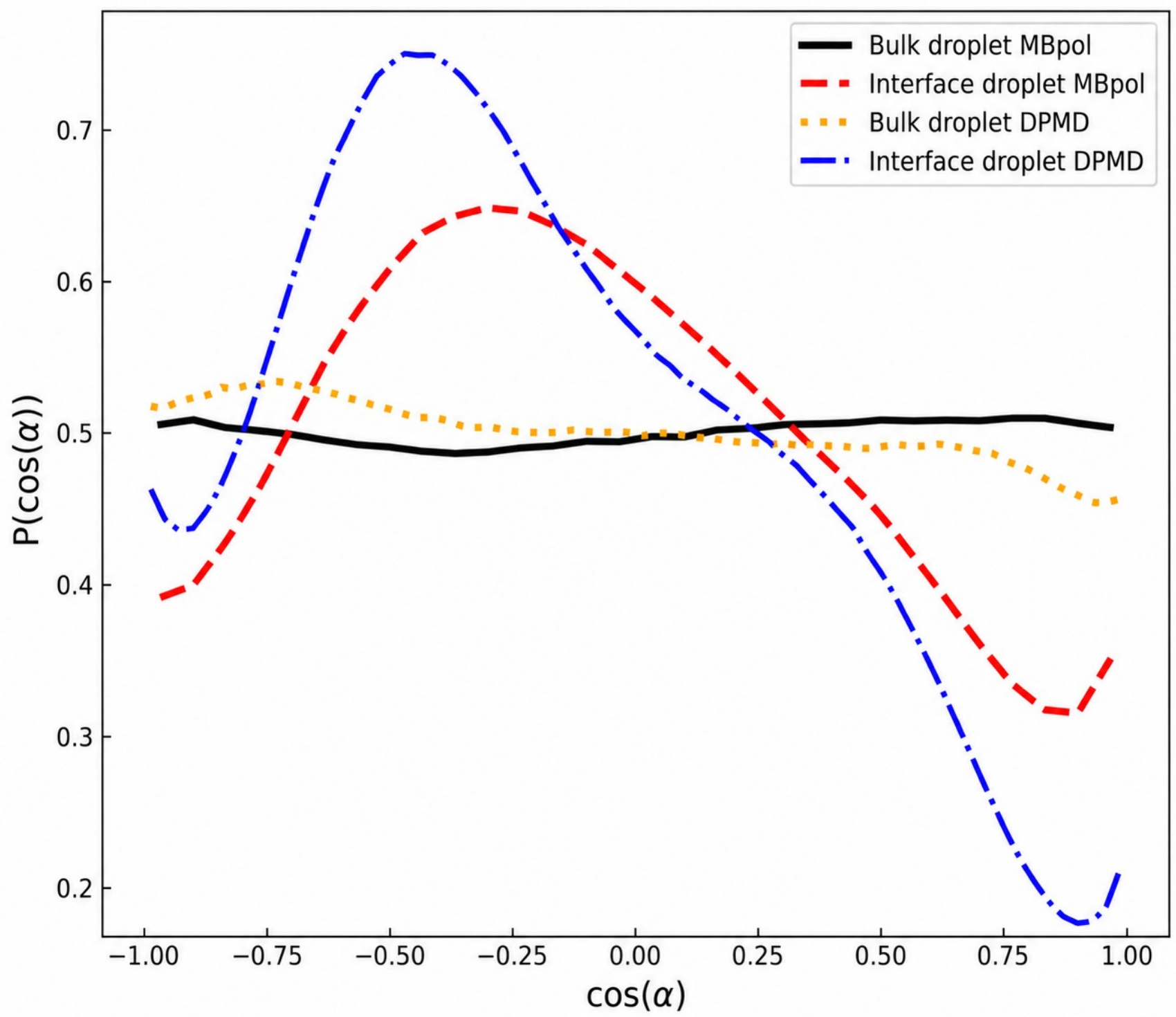

Figure S18: Orientation distributions of molecular dipole moments P(cos(α)) with respect to the Willard-Chandler surface normal determined in the bulk and at the interfaces of a nanodroplet system composed of 512 H_2O molecules and simulated via MB-pol(2023) and the short-range deep potential model employed in our work (see legend).

interactions in the charged systems. This way, we have also examined separately the bulk-like regions of all the neutral, protonated, and hydroxide-containing droplets considered in this work. In every case, the corresponding cosα distribution remains nearly uniform within the droplet interior, and the orientational anisotropy remains localized within the interfacial region. In Fig. S19, we report the distributions of the angles formed by the water dipoles in the neutral, and singly charged systems. We therefore find no evidence of the long-distance, bulk-spanning dipolar alignment identified as a characteristic artifact of pathological short-range potentials. Nevertheless, we acknowledge that the finite systems' sizes considered in this work may limit sensitivity to very long-wavelength electrostatic correlations and, therefore, cannot entirely rule out the presence of subtle long-range effects at larger scales.

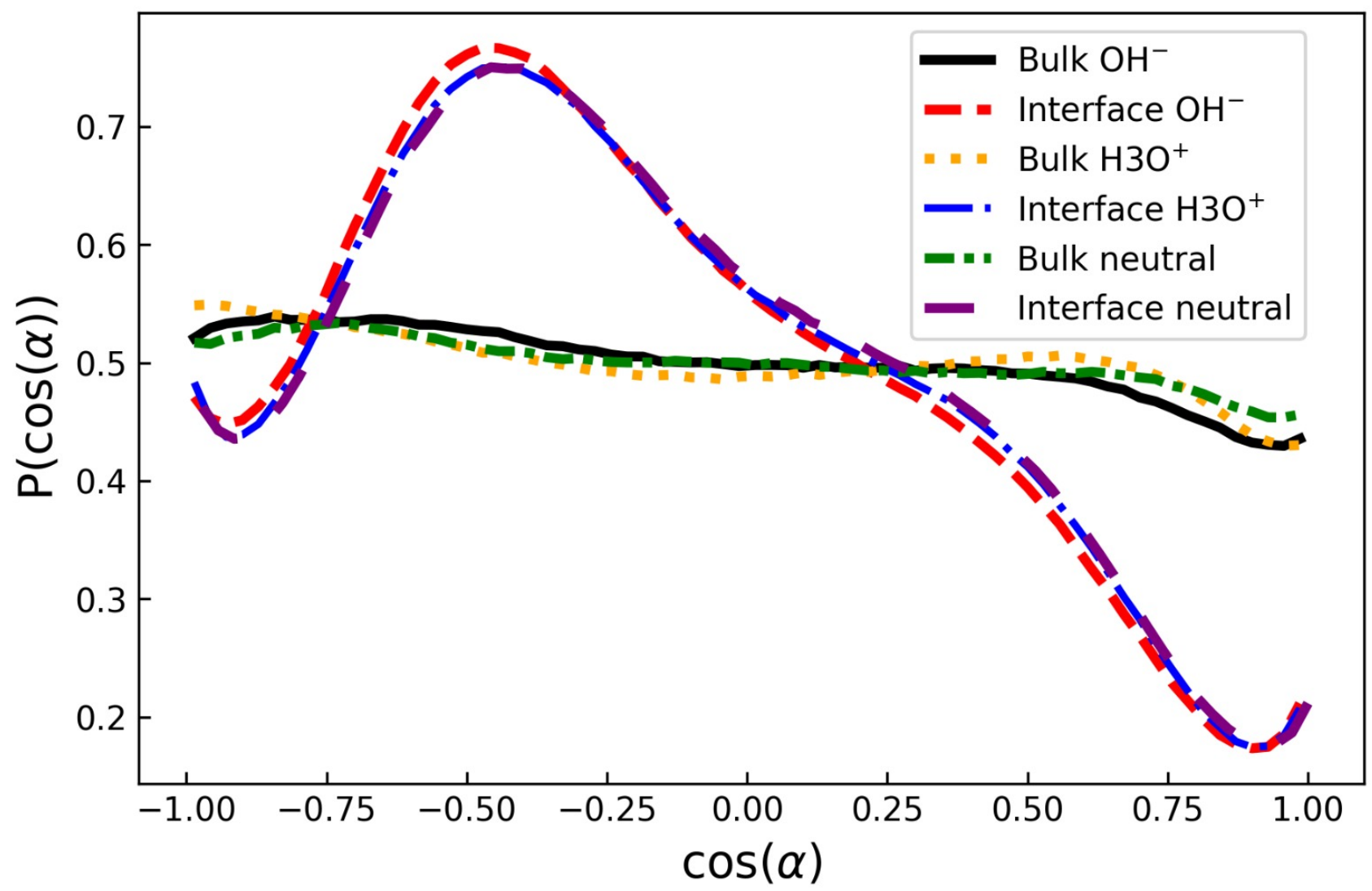

Figure S19: Orientation distributions of molecular dipole moments P(cos(α)) with respect to the Willard-Chandler surface normal determined in the bulk and at the interfaces of a neutral nanodroplet system composed of 512 H_2O molecules, a nanodroplet composed of 511 H_2O and 1 OH^-, and a nanodroplet composed of 511 H_2O and 1 H_3O^+ simulated via the short-range deep potential model employed in our work (see legend).

Validation Against a Long-Range Deep Potential Model

Additionally to the already presented analysis on the dipolar behavior, we have also executed the most direct test of whether the short-range representation introduces a systematic bias for the droplet configurations examined here by comparing energies and forces prediction with respect to a long-range DP (DPLR) model. We have therefore evaluated both the production DP and an existing DPLR model[19] on the same 2500 configurations: 500 configurations from each of the five main systems, comprising neutral droplets containing 256, 512, and 1024 water molecules and 512-molecule droplets containing one excess hydronium or hydroxide ion.

To accommodate the periodic implementation of DPLR while minimizing direct interactions between periodic images, each configuration was translated so that its atomic centroid coincided with the center of a 135 Å cubic cell. This cell length is approximately three times the largest Cartesian span found across the entire dataset (43.60 Å), providing a nominal empty-space margin of approximately 45.7 Å between the droplet and each cell face. After

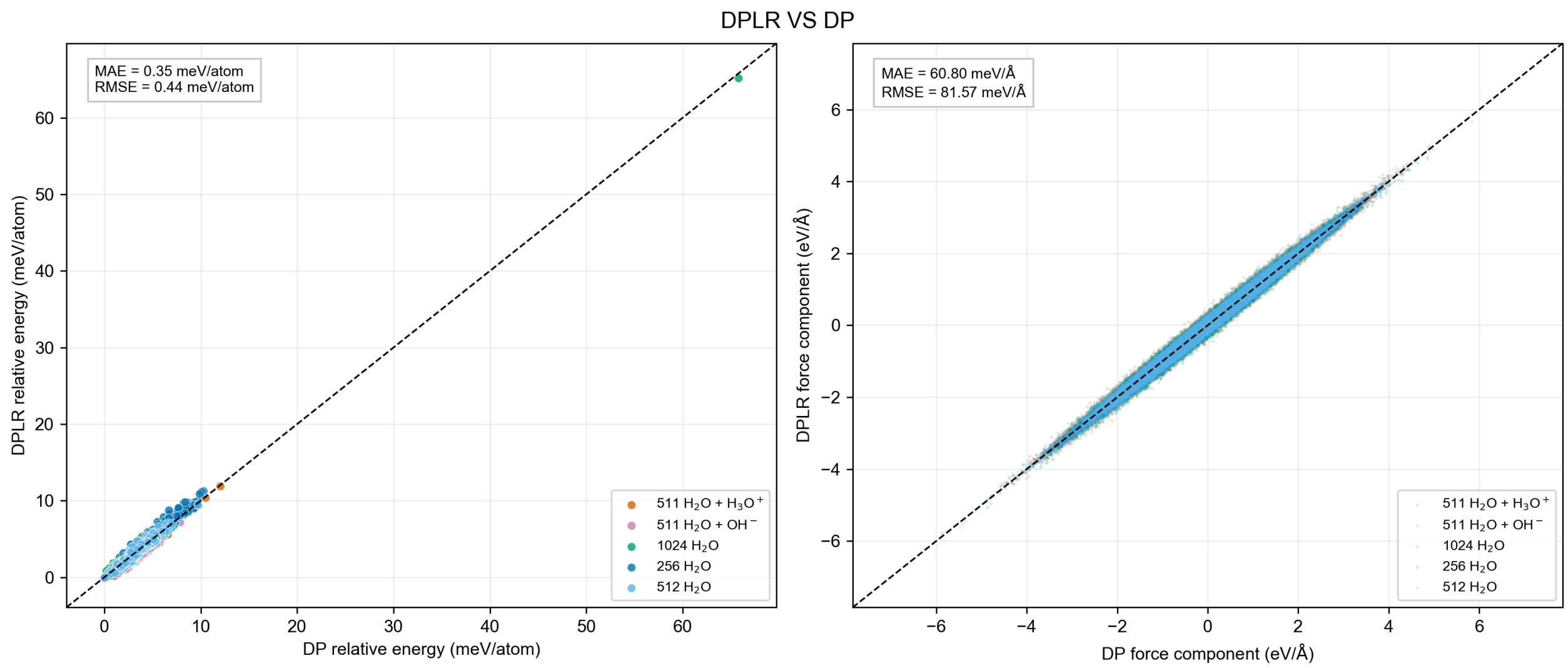

Figure S20: Comparison between the explicit long-range DPLR model and the short-range DP model on identical configurations from neutral droplets of 256, 512, and 1024 water molecules and from hydronium- and hydroxide-containing 512-molecule droplets. Left: relative energies per atom. Right: Cartesian force components. The dashed line is y = x.

applying the same energy-shifting procedure, DP and DPLR yield closely correlated relative energies and force components across all five systems. As shown in Fig. S20, the energy differences have an MAE of 0.35 meV/atom and an RMSE of 0.44 meV/atom, while the force-component differences have an MAE of 60.80 meV/Å and an RMSE of 81.57 meV/Å. Importantly, no systematic increase in the deviations is observed with droplet size or upon inclusion of hydronium or hydroxide ions. Thus, within the configurational domain sampled in this work, introducing the explicit long-range contribution does not produce a systematic alteration of the relative energies or atomic forces.

Charge Transfer Analysis

To quantify the electronic charge fluctuations associated with each atom, we analyzed the electron density stemming from self-consitent-field calculations of molecular snapshots extracted from deep potential molecular dynamics simulations of all the investigated systems using an in-house C++ code. The electronic density was partitioned using a van der Waals weighted Voronoi tessellation of space. In this scheme, each voxel of the real-space grid is

assigned to the atom that minimizes the weighted distance

$$d_a = \frac{|\mathbf{r} - \mathbf{R}_a|}{r_a^{\text{vdW}}}, \tag{1}$$

where $\mathbf{R}_a$ is the atomic position and r_a^{vdW} is the van der Waals radius of atom a. This weighted partitioning prevents the systematic over-assignment of electronic density to smaller atoms and provides a physically meaningful atomic decomposition of the density.

The number of electrons associated with each atom was obtained by integrating the electron density over the corresponding Voronoi region

$$N_a = \int_{\Omega_a} \rho(\mathbf{r})\, d\mathbf{r}, \tag{2}$$

where Ω_a denotes the Voronoi cell associated with atom a.

The charge fluctuation for each atom was then defined as the deviation of the instantaneous electron population from the average value for atoms of the same chemical species,

$$\delta q_a = N_a - \langle N_a \rangle_{Z_a}, \tag{3}$$

where $\langle N_a \rangle_{Z_a}$ is the mean number of electrons associated with atoms of atomic number Z_a within the configuration. This quantity can be interpreted as the charge fluctuation induced by the local molecular environment and intermolecular interactions.[20]

References

(1) Zhang, P.; Xu, X. Propensity of Water Self-Ions at Air(Oil)–Water Interfaces Revealed by Deep Potential Molecular Dynamics with Enhanced Sampling. *Langmuir* **2025**, *41*, 3675–3683, PMID: 39882949.

(2) Zeng, J. et al. DeePMD-kit v2: A Software Package for Deep Potential Models. *J. Chem. Phys.* **2023**, *159*, 054801.

(3) Zhao, Y.; Truhlar, D. G. The M06 suite of density functionals for main group thermochemistry, thermochemical kinetics, noncovalent interactions, excited states, and transition elements: two new functionals and systematic testing of four M06-class functionals and 12 other functionals. *Theoretical Chemistry Accounts* **2008**, *120*, 215–241.

(4) Willard, A. P.; Chandler, D. Instantaneous Liquid Interfaces. *The Journal of Physical Chemistry B* **2010**, *114*, 1954–1958.

(5) Pezzotti, S.; Galimberti, D. R.; Gaigeot, M.-P. 2D H-Bond Network as the Topmost Skin to the Air–Water Interface. *The Journal of Physical Chemistry Letters* **2017**, *8*, 3133–3141, PMID: 28644626.

(6) Luzar, A.; Chandler, D. Hydrogen-bond kinetics in liquid water. *Nature* **1996**, *379*, 55–57.

(7) de la Puente, M.; Laage, D. Impact of interfacial curvature on molecular properties of aqueous interfaces. *The Journal of Chemical Physics* **2024**, *160*, 234504.

(8) Hao, H.; Leven, I.; Head-Gordon, T. Can electric fields drive chemistry for an aqueous microdroplet? *Nature Communications* **2022**, *13*, 280.

(9) Yesibolati, M. N.; Laganà, S.; Sun, H.; Beleggia, M.; Kathmann, S. M.; Kasama, T.; Mølhave, K. Mean Inner Potential of Liquid Water. *Phys. Rev. Lett.* **2020**, *124*, 065502.

(10) Perdew, J. P.; Burke, K.; Ernzerhof, M. Generalized gradient approximation made simple. *Physical Review Letters* **1996**, *77*, 3865.

(11) Grimme, S.; Antony, J.; Ehrlich, S.; Krieg, H. A consistent and accurate ab initio parametrization of density functional dispersion correction (DFT-D) for the 94 elements H-Pu. *J. Chem. Phys.* **2010**, *132*, 154104.

(12) Grimme, S.; Ehrlich, S.; Goerigk, L. Effect of the damping function in dispersion corrected density functional theory. *J. Comput. Chem.* **2011**, *32*, 1456–1465.

(13) Krack, M. Pseudopotentials for H to Kr optimized for gradient-corrected exchange-correlation functionals. *Theoretical Chemistry Accounts* **2005**, *114*, 145–152.

(14) Kathmann, S. M.; Kuo, I.-F. W.; Mundy, C. J.; Schenter, G. K. Understanding the Surface Potential of Water. *The Journal of Physical Chemistry B* **2011**, *115*, 4369–4377, PMID: 21449605.

(15) Sun, J.; Ruzsinszky, A.; Perdew, J. P. Strongly Constrained and Appropriately Normed Semilocal Density Functional. *Physical Review Letters* **2015**, *115*, 036402.

(16) Furness, J. W.; Kaplan, A. D.; Ning, J.; Perdew, J. P.; Sun, J. Correction to "Accurate and Numerically Efficient r2SCAN Meta-Generalized Gradient Approximation". *J. Phys. Chem. Lett.* **2020**, *11*, 9248.

(17) Niblett, S. P.; Galib, M.; Limmer, D. T. Learning intermolecular forces at liquid–vapor interfaces. *The Journal of Chemical Physics* **2021**, *155*, 164101.

(18) Zhu, X.; Riera, M.; Bull-Vulpe, E. F.; Paesani, F. MB-pol(2023): Sub-chemical Accuracy for Water Simulations from the Gas to the Liquid Phase. *Journal of Chemical Theory and Computation* **2023**, *19*, 3551–3566, PMID: 37249505.

(19) Zhang, P.; Xu, X. Modulation of Electric Field and Interface on Competitive Reaction Mechanisms. *Journal of Chemical Theory and Computation* **2025**, *21*, 6584–6593.

(20) Ben-Amotz, D. Electric buzz in a glass of pure water. *Science* **2022**, *376*, 800–801.
